# Wall teichoic acids regulate peptidoglycan synthesis to maintain rod shape in *Bacillus subtilis*

**DOI:** 10.1038/s41564-026-02368-6

**Published:** 2026-05-26

**Authors:** Felix Barber, Zarina Akbary, Zhe Yuan, Jacob Biboy, Waldemar Vollmer, Enrique R. Rojas

**Affiliations:** 1 Center for Genomics and Systems Biology, New York University, New York, NY USA; 2 Department of Biology, New York University, New York, NY USA; 3 Center for Bacterial Cell Biology, Newcastle University, Newcastle upon Tyne, UK; 4 Institute for Molecular Bioscience, The University of Queensland, Brisbane, Queensland Australia

**Keywords:** Cellular microbiology, Molecular biophysics

## Abstract

Rod-shaped bacteria such as *Bacillus subtilis* achieve their shape by using Rod complexes to synthesize anisotropic peptidoglycan and by limiting isotropic peptidoglycan synthesis by PBP1. Wall teichoic acids are also required for rod shape, but their role is unclear. Here we use single-cell microfluidics and microscopy to show that wall teichoic acids promote rod shape by preventing the formation of nanoscopic pores in the *B. subtilis* cell wall. Wall teichoic acid depletion led to pore formation within minutes, coinciding with a rapid increase in PBP1-mediated peptidoglycan synthesis, which became essential for growth, and transient arrest of Rod complexes before the onset of amorphous growth. A synthetically lethal cell wall hydrolase, LytE, also became essential during wall teichoic acid depletion, meaning that PBP1 and LytE cooperatively execute amorphous growth in the absence of teichoic acids. Our results show that wall teichoic acids maintain cell shape by preventing cell wall pore formation, thereby promoting Rod complex activity and preventing PBP1 activity.

## Abstract (web summary)

Wall teichoic acids confer rod shape in the Gram-positive model organism *Bacillus subtilis* by occluding pores in the cell wall, thereby preventing runaway isotropic (non-oriented) peptidoglycan synthesis.

## Main

**Fig. 1:**
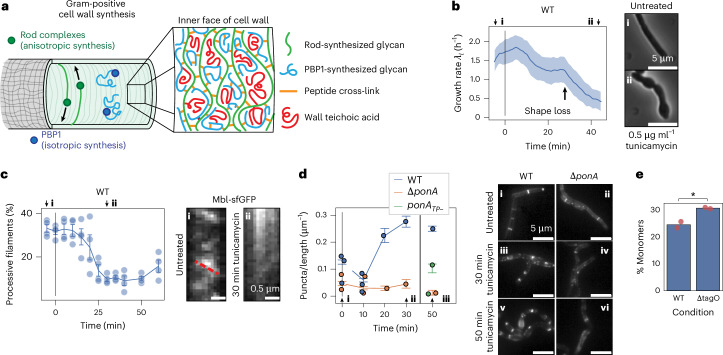
Inhibiting wall teichoic acid synthesis decreases Rod complex activity before cell shape loss. **a**, Schematic of Gram-positive cell wall synthesis. **b**, Left: cell length growth rate ${{\rm{\lambda }}}_{l}=\frac{1}{l}\frac{{\rm{d}}l}{{\rm{d}}t}$ for wild-type cells during 0.5 μg ml^−1^ tunicamycin treatment. Data presented as smoothed median ± s.d.; 2,048 cell tracks, 3 biological replicates. Right: micrographs taken (**i**) before and (**ii**) after 45 min tunicamycin treatment. Scale bars, 5 μm. **c**, Left: percentage of processive Mbl filaments during a timecourse of 0.5 μg ml^−1^ tunicamycin treatment. Data are presented as average across biological replicates ±s.e.m. Dots show biological replicates; 62,901 filament tracks, 4 biological replicates. Right: representative fluorescence kymographs of Mbl motion across the cell waist in (**i**) LB and (**ii**) after 30 min tunicamycin treatment. Red dashed line follows a processive Mbl filament. Scale bars, 0.5 μm. **d**, Left: mean fluorescent puncta per micron of cell length for wild-type, Δ*ponA* and *ponA*_*TP−*_ cells during tunicamycin treatment, labelled with fluorescent D-amino acids. Error bars show 95% confidence intervals; 2,017 segmentations from 4 biological replicates (wild type), 857 segmentations from 3 biological replicates (Δ*ponA*) and 257 cells from 2 biological replicates (*ponA*_*TP−*_). All timepoints show statistical significance of differences between cell types (two-sided Student’s *t*-test): 0 min: *p* = 1 × 10^−35^ (wild-ty*p*e vs Δ*ponA*); 10 min: *p* = 8 × 10^−9^ (wild-ty*p*e vs Δ*ponA*); 20 min: *p* = 3 × 10^−34^ (wild-ty*p*e vs Δ*ponA*); 30 min: *p* = 1 × 10^−25^ (wild-ty*p*e vs Δ*ponA*); 50 min: *p* = 4 × 10^−24^ (wild-type vs Δ*ponA*), *p* = 3 × 10^−48^ (wild-type vs *ponA*_*TP−*_), *p* = 4 × 10^−4^ (Δ*ponA* vs *ponA*_*TP−*_). Dots show biological replicates. Measurements at 50 min tunicamycin treatment used a lower exposure time. Right: micrographs of (**i**,**iii**,**v**) wild-type and (**ii**,**iv**,**vi**) Δ*ponA* cells at (**i**,**ii**) 0 min, (**iii**,**iv**) 30 min and (**v**,**vi**) 50 min of treatment. Micrographs are identically saturated across cell types. Scale bars, 5 μm. **e**, Percentage of peptidoglycan subunits in monomers. Plotted is the average across 2 biological replicates (dots). Statistical significance calculated using two-sided Student’s *t*-test, *p = 0.03. Source data

The bacterial cell wall is a solid polymeric macromolecule that is the target of our best antibiotics because it protects cells from lysis in many contexts, particularly during osmotic shock^1^ and pathogenesis^2^. The cell wall also prescribes cell shape (Fig. 1a), which affects many aspects of cellular physiology^3–5^; a pervasive shape across bacteria is the bacillus or ‘rod’^6^. The cell wall consists primarily of peptidoglycan composed of glycan polymers that are covalently cross-linked by short peptides. In Gram-positive bacteria, peptidoglycan is also covalently modified with wall teichoic acids—anionic polymers of sugar alcohol–phosphate subunits (hereafter, ‘teichoic acids’, distinct from the membrane-anchored lipoteichoic acids)^7,8^. Teichoic acids are not essential^9,10^; however, inhibiting their synthesis causes *Bacillus subtilis* and *Listeria monocytogenes* to grow slowly^10^ and amorphously^9,11^. How teichoic acids enable rod-shaped morphogenesis is unknown^9–11^.

During rod-shaped cell growth, peptidoglycan is synthesized through two molecular mechanisms. First, the essential, multiprotein Rod complexes synthesize anisotropic peptidoglycan that structurally reinforces the cell wall along its circumference (Fig. 1a), providing the mechanical basis for rod shape^12,13^. Anisotropic peptidoglycan synthesis results from the processive circumferential motion of Rod complexes along the plasma membrane^14–16^. In *B. subtilis*, this motion is oriented by actin homologues MreB, Mbl and MreBH^17–19^, and is driven by peptidoglycan synthesis itself^14^. Circumferential motion, therefore, is a direct indicator for Rod complex activity^20,21^. Within the Rod complex, peptidoglycan polymerization is catalysed by the transglycosylase RodA^22^, drawing from lipid II precursors in the plasma membrane, while cross-linking of nascent polymers to acceptor peptides within the existing cell wall is catalysed by the transpeptidases PBP2A and PBPH^23,24^ (‘Class B’ penicillin-binding proteins, PBPs).

The second mechanism is the non-essential, multidomain enzyme PBP1 (a ‘Class A’ PBP, encoded by *ponA*^25^) that synthesizes isotropic peptidoglycan (Fig. 1a). PBP1 is thought to repair pores in the cell wall that arise during the rapid cell-wall turnover required for cell growth^23,26,27^. Because Rod complexes synthesize peptidoglycan anisotropically and PBP1 synthesizes it isotropically, the balance of their expression determines cell width^12^, and overexpression of PBP1 causes semi-amorphous growth^12^.

Teichoic acids influence diverse cellular phenotypes including antimicrobial resistance^28,29^, protein localization within the cell envelope^30^, pathogenesis^31^, cation homeostasis^32,33^ and autolysis^34–37^. These correlations, however, do not explain the dependence of cell shape on teichoic acids according to the Rod complex–PBP1 model. Similarly, inhibiting late reactions of teichoic acid synthesis sequesters the lipid precursor undecaprenyl phosphate and blocks peptidoglycan synthesis^38^, but this does not explain why deleting *tagO*, which encodes the enzyme that catalyses the first committed reaction of teichoic acid synthesis^19,38,9^, or pharmacological inhibition of TagO, affects cell shape. One possibility is that Rod complexes are directly activated by teichoic acids, biochemically^39^. This model is consistent with the observations that teichoic acid synthases and the peptidoglycan synthesis machinery both exhibit punctate spatial patterning^40^, and that the syntheses of the two materials are co-local in both *B. subtilis*^41^ and *Streptococcus pneumoniae*^42^. However, teichoic acid synthases do not move circumferentially like Rod complexes^19^, and Rod complexes move processively even in amorphous cells depleted of TagO^19,38^. Given these conflicting data, there is no known mechanistic interaction between teichoic acids and either of the two peptidoglycan biosynthesis pathways.

## Results

### Depleting wall teichoic acids slows growth before shape loss

**Extended Data Fig. 1:**
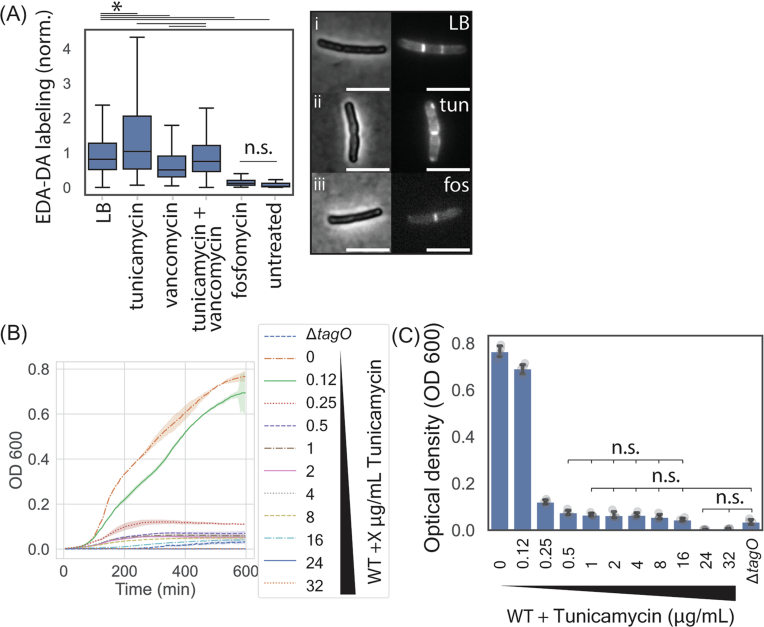
Tunicamycin specifically inhibits tagO at low concentrations. **(a)** Box plots showing intensity of clickable ethynyl-D-alanine D-alanine, which is incorporated into peptidoglycan precursors cytosolically^48^, within the cell envelope for cells co-treated with the drugs shown (*Methods*), normalized against average staining in LB. Box lines show quartiles, whiskers show spread of data (excluding outliers for ease of presentation). Tunicamycin increased staining both in LB, and in vancomycin-treated cells, consistent with a rerouting of precursors to peptidoglycan synthesis during TagO inhibition. Fosfomycin treatment arrests peptidoglycan precursor synthesis at the inner face of the plasma membrane so is a negative control. Untreated cells were not exposed to EDA-DA to control for off-target click labeling. n = 1,533 cells (LB, 5 biological replicates), n = 1,063 cells (tunicamycin, 5 biological replicates), n = 810 cells (tunicamycin + vancomycin, 3 biological replicates), n = 1,081 cells (vancomycin, 4 biological replicates), n = 555 cells (fosfomycin control, 4 biological replicates), and n = 473 cells (untreated, 4 biological replicates). All differences between conditions were significant (one-way ANOVA followed by Tukey’s honestly significant difference (HSD) post-hoc test, P < 0.01), with the exception of comparisons between fosfomycin-untreated and between LB-tunicamycin+vancomycin. Inset: phase contrast and fluorescence micrographs of cells from (i) LB, (ii) tunicamycin and (iii) fosfomycin conditions. Fluorescence micrographs are identically saturated. Scale bars 5 μm. **(b-c)** Low tunicamycin concentrations inhibit growth no more than does deletion of *tagO*. **(b)** Growth curves of cells grown with increasing tunicamycin concentrations in bulk culture. Plotted are median OD600 measurements across 4 technical replicates (individual replicates shown as dots) for one representative biological replicate out of three. Error bars show standard deviation across technical replicates. **(c)** Saturating culture density plotted for OD600 data from (A). Plotted is the average across 4 technical replicates (individual replicates shown as dots) for one representative biological replicate out of three. Error bars show 95% confidence intervals based on bootstrap analysis. Complete growth arrest consistently occurs at either 16 μg/mL or 24 μg/mL tunicamycin. All differences are significant except those specifically marked. Statistical significance tested using one-way ANOVA followed by Tukey’s honestly significant difference (HSD) post-hoc test, P < 0.01.

**Extended Data Fig. 2:**
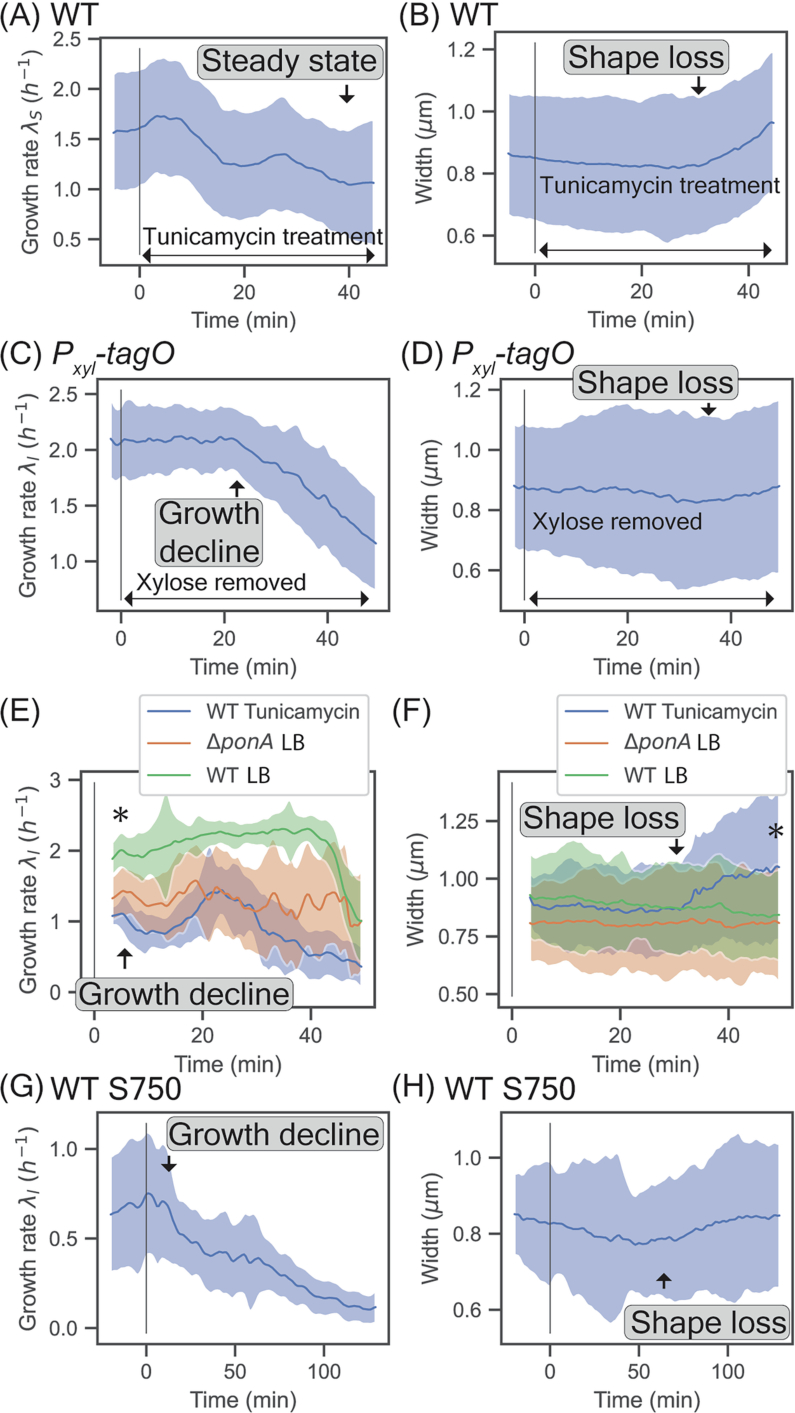
Elongation slows prior to shape loss during wall teichoic acid depletion. Solid lines show smoothed population medians, error bars show standard deviations across cells. Black lines show onset of perturbation (tunicamycin treatment, inducer depletion or cell spotting onto agarose pads). **(a)** Cell surface area growth rate ${{\rm{\lambda }}}_{S}=\frac{1}{S}\frac{{dS}}{{dt}}$ for wild-type cells during 0.5 μg/mL tunicamycin treatment. 1,927 cell tracks, 3 biological replicates. **(b)** Cell width of wild-type cells during tunicamycin timecourse. Increase in width corresponds to gradual loss of cell shape. 3,737 cell tracks, 3 biological replicates. **(c-d)** Cell properties during growth response of *P*_*xylA*_*-tagO* cells to xylose depletion. **(c)** Cell length growth rate. 1,222 cell tracks, 3 biological replicates. **(d)** Cell width. 1,968 cell tracks, 3 biological replicates. **(e-f)** Cell growth and shape response to tunicamycin treatment on agarose pads. **(e)** Cell length growth rate. WT LB: 204 cell tracks, 4 biological replicates. WT tunicamycin: 160 cell tracks, 3 biological replicates. Δ*ponA* LB: 93 cell tracks, 3 biological replicates. Tunicamycin slows growth immediately, with oscillations, followed by a secondary decline at 30 min coincident with shape loss. WT cells grown in LB alone showed an abrupt growth decline at 45 min with no shape loss. This late-stage decline was not observed for the slower-growing Δ*ponA* cells, consistent with local nutrient depletion in these ~1mm-thin agarose pads. WT LB and WT tunicamycin significance tested using two-sided Student’s *t*-test at first measured timepoint, *p* = 4e-20. **(f)** Cell width. WT LB: 535 cell tracks, 4 biological replicates. WT tunicamycin: 432 cell tracks, 3 biological replicates. Δ*ponA* LB: 214 cell tracks, 3 biological replicates. WT LB and WT tunicamycin statistical significance tested using two-sided Student’s *t*-test at last measured timepoint (63 min), *p* = 5e-6. **(g-h)** Time course of 0.5 μg/mL tunicamycin exposure for WT cells grown in S750 media. **(g)** Cell length growth rate. 249 cell tracks, 2 biological replicates. **(h)** Cell width. 307 discrete cell tracks, 2 biological replicates.

*Bacillus subtilis* mutants that lack teichoic acids (Δ*tagO*) grow amorphously and ~4 times slower than wild-type cells in bulk liquid culture^10^. To interrogate this dependence at the single-cell level, we measured the dynamics of growth rate and cell shape upon teichoic acid depletion by using microfluidics to acutely treat exponentially growing, wild-type *B. subtilis* cells with the small-molecule tunicamycin (Fig. 1b), which specifically inhibits TagO at low concentrations^29,43–45^ (Extended Data Fig. 1a–c). Approximately 10 min after tunicamycin treatment, the median growth rate decreased from its steady-state value in rich medium (LB), plateaued temporarily and then decreased a second time until it reached its steady-state value in tunicamycin (Fig. 1b and Extended Data Fig. 2a). Cells retained their untreated rod shape until the second decrease in elongation rate when they began to widen via an intermediate ‘dumbbell’ morphology (Fig. 1b inset) before eventually losing the rod shape altogether (Extended Data Fig. 2b). This transient ‘dumbbell’ phenotype may reflect local reinforcement of the cell wall by septa (Supplementary Fig. 1)^10^. Amorphous cells grew indefinitely (Supplementary Video 1). We observed a similar decline in the single-cell growth rate before the loss of cell shape when we acutely inhibited *tagO* transcription (Extended Data Fig. 2c,d), during tunicamycin treatment on agarose pads (Extended Data Fig. 2e,f), and during tunicamycin treatment in minimal media (S750; Extended Data Fig. 2g,h and Methods).

### Depleting wall teichoic acids reduces Rod complex activity

**Extended Data Fig. 3:**
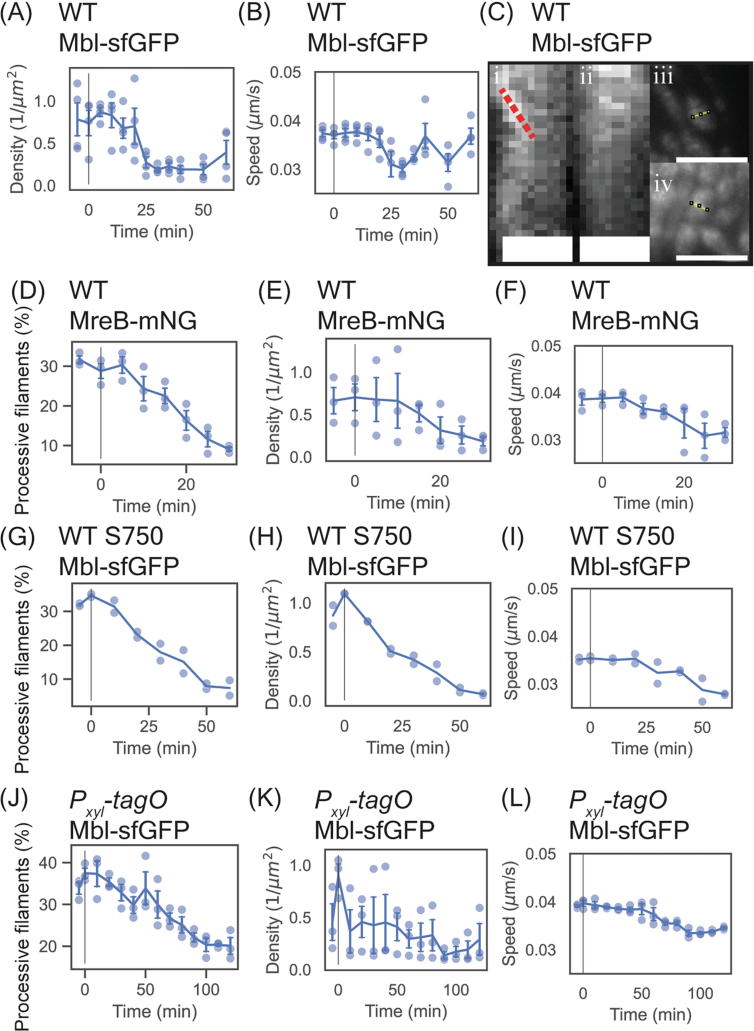
Processive Rod complex motion declines during wall teichoic acid depletion. **(a-b)** Mbl-sfGFP activity time course for wild-type cells during 0.5 μg/mL tunicamycin treatment. Data are presented as average across biological replicates ± SEM, with individual biological replicates as dots. 62,901 filament tracks, 4 biological replicates. **(a)** Spatial density of processive filaments. **(b)** Average speed of processive filaments. **(c)** (i-ii) Representative kymographs from TIRF imaging of Mbl-sfGFP cells after 60 min tunicamycin treatment. Dotted line follows a processive Mbl filament. Scale bars 1 μm. (iii-iv) TIRF micrographs showing whole cells during a single timepoint of the kymographs in (i-ii) respectively. Yellow lines show kymograph traces. Scale bars 5 μm. **(d–f)** MreB-mNeonGreen activity time course for wild-type cells during 0.5 μg/mL tunicamycin treatment. Data are presented as average across biological replicates ± SEM, with individual biological replicates as dots. 30,805 filament tracks, 3 biological replicates. **(d)** Percentage of processive filaments. **(e)** Spatial density of processive filaments. **(f)** Average speed of processive filaments. **(g-i)** Mbl-sfGFP activity time course for wild-type cells grown in S750 media during 0.5 μg/mL tunicamycin treatment. Data are presented as average across biological replicates, with individual biological replicates as dots. 9,859 filament tracks, 2 biological replicates. **(g)** Percentage of processive filaments. **(h)** Spatial density of processive filaments. **(i)** Average speed of processive filaments. **(j–l)** Mbl-sfGFP activity time course of of *P*_*xylA*_*-tagO* cells during xylose depletion. Data are presented as average across biological replicates ± SEM, with individual biological replicates as dots. 55,542 filament tracks, 3 biological replicates. **(j)** Percentage of processive filaments. **(k)** Spatial density of processive filaments. **(l)** Average speed of processive filaments.

Although it was previously reported that inhibiting teichoic acid synthesis does not prevent Rod complex motion during steady state, amorphous cell growth^19,38^, we hypothesized that the loss of cell shape results from a quantitative or transient effect of teichoic acid depletion on Rod complex activity. To test this, we measured the dynamics of Rod complex motion upon tunicamycin treatment by using total internal reflection fluorescence microscopy to track a fusion of superfolder GFP and Mbl^14,20,21^, a protein scaffold of the Rod complex. As we hypothesized, tunicamycin caused an acute decrease in the percentage of Rod complexes that moved processively (Fig. 1c), in the spatial density of processive Rod complexes (Extended Data Fig. 3a), and in Rod complex speed (Extended Data Fig. 3b). These decreases were coincident with the first decline in cellular growth rate (Fig. 1b). Following the second decrease in growth rate (and loss of cell shape; Fig. 1b), we observed a small but reproducible recovery of Rod complex motion (Fig. 1c) against a higher background of diffuse Mbl-sfGFP fluorescence than we observed in untreated cells (Extended Data Fig. 3c and Supplementary Videos 2–7), consistent with the previous observation of Rod complex motion in Δ*tagO* mutants^19^. Tunicamycin also inhibited Rod complex motion as tracked via another Rod complex scaffold (MreB-mNeonGreen; Extended Data Fig. 3d–f), and inhibited the Rod complexes of slow-growing cells in minimal media (Extended Data Fig. 3g–i and Methods). Finally, similar to tunicamycin treatment, transcriptional inhibition of *tagO* expression caused a decrease in each metric for Rod complex activity (Extended Data Fig. 3j–l). The decrease was more gradual for transcriptional inhibition, which we expected since in this case TagO enzymes must be diluted by growth before teichoic acids are depleted.

**Extended Data Fig. 4:**
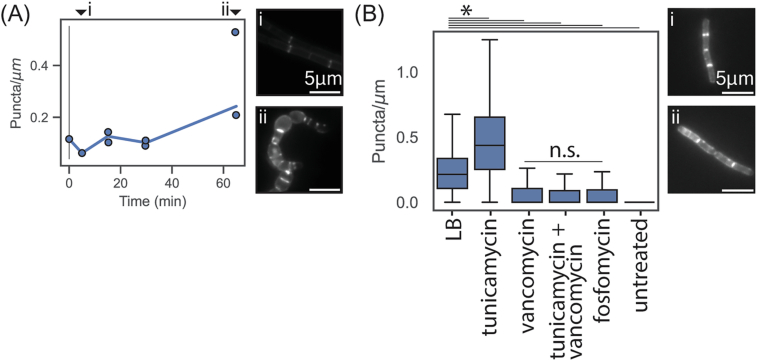
Wall teichoic acid depletion causes punctal peptidoglycan synthesis. **(a)** Fluorescent puncta per cell (normalized to cell length) for *P*_*xylA*_*-tagO* cells during xylose depletion, labeled with fluorescent D-amino acids. Black line shows xylose washout. Plotted is the average over 680 segmented cells from 2 biological replicates. Inset: micrographs at (i) 5 min and (ii) 65 min following xylose depletion. Micrographs are identically saturated across timepoints. **(b)** Box plots showing EDA-DA AF488 click-labeled puncta per cell (normalized to cell length) for wild-type cells during co-treatments with antibiotics shown (*Methods*). Box lines show quartiles, whiskers show spread of data (excluding outliers for ease of presentation). n = 1,533 cells (LB, 5 biological replicates), n = 1,063 cells (tunicamycin, 5 biological replicates), n = 810 cells (tunicamycin + vancomycin, 3 biological replicates), n = 1,081 cells (vancomycin, 4 biological replicates), n = 555 cells (fosfomycin control, 4 biological replicates), and n = 473 cells (unstained, 4 biological replicates). All differences between conditions were significant by one-way ANOVA followed by Tukey’s HSD post-hoc test, *p*-value below computational detection limit (LB v. tunicamycin, LB v. vancomycin, LB v. tunicamycin + vancomycin, LB v. fosfomycin, LB v. untreated), with the exception of comparisons between vancomycin, tunicamycin+vancomycin and fosfomycin, which did not show significance. Inset: fluorescent micrographs of AF488 labeling in cells cultured with (i) LB alone and (ii) tunicamycin. Micrographs are identically saturated across conditions. Scale bars 5 μm.

Since Rod complexes synthesize peptidoglycan uniformly across the cell length, we hypothesized that this distributed synthesis would be perturbed upon the Rod complex arrest associated with teichoic acid depletion. To test this, we measured the localization of peptidoglycan synthase activity. First, we pulsed exponentially growing cells with the fluorescent D-amino acid 7-hydroxycoumarin-3-carboxylic acid-amino-D-alanine (HADA), which is incorporated into peptidoglycan by transpeptidases^46,47^, for 5 min at various times after tunicamycin treatment. Whereas in untreated cells HADA was incorporated uniformly across the cell wall, tunicamycin treatment caused HADA to be incorporated as aberrant puncta (Fig. 1d), consistent with a loss of distributed Rod complex synthesis. Tunicamycin had the same effect on the dipeptide D-amino acid ethynyl-D-alanine D-alanine (EDA-DA), which is incorporated into peptidoglycan precursors in the cytoplasm^48^ (Extended Data Fig. 4b). Finally, transcriptional inhibition of *tagO* also caused punctal HADA incorporation (Extended Data Fig. 4a).

**Extended Data Fig. 5:**
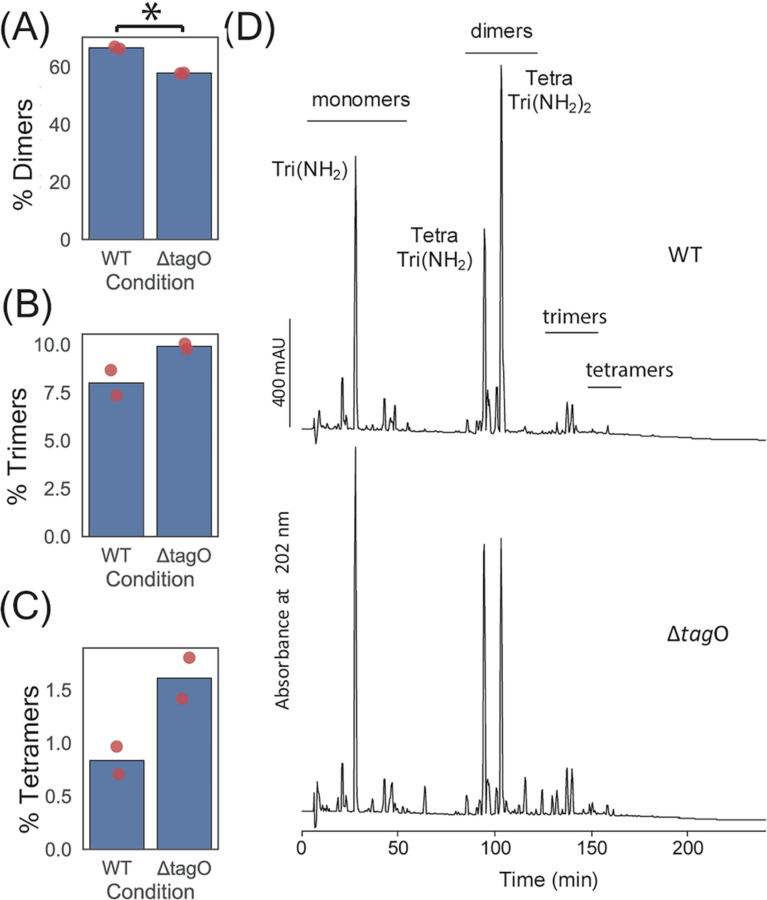
Constitutive wall teichoic acid depletion decreases dimeric peptidoglycan crosslinks. **(a-c)** Percentage peptidoglycan subunits in **(a)** dimers, **(b)** trimers and **(c)** tetramers. Plotted is the average across two biological replicates. Individual datapoints shown in red. Statistical significance calculated using Student’s *t*-test, P < 0.05. Differences for (B) and (C) are not statistically significant by this metric but reflect visible shifts in high time peaks that are consistent between samples (see (D)). **(d)** High-performance liquid chromatograms of reduced muropeptide samples isolated from wild-type and Δ*tagO* cells. Elution regions of the uncross-linked monomers and cross-linked dimers, trimers and tetramers are shown, and the three main muropeptides are labelled. Tri(NH_2_), GlcNAc-MurNAc(red)-L-Ala-D-iGlu-mDap (1 amidation); TetraTri(NH2), GlcNAc-MurNAc(red)-L-Ala-D-iGlu-mDap-D-Ala-mDap-D-iGlu-L-Ala-(GlcNAc)MurNAc(red) (1 amidation); TetraTri(NH2)2, GlcNAc-MurNAc(red)-L-Ala-D-iGlu-mDap-D-Ala-mDap-D-iGlu-L-Ala-(GlcNAc)MurNAc(red) (2 amidations). GlcNAc, N-acetylglucosamine; MurNAc(red), N-acetylmuramitol; L-Ala, L-alanine; D-iGlu, D-isoglutamic acid; mDap, meso-diaminopimelic acid; D-Ala, D-alanine.

Since Rod complexes are the primary mode of peptidoglycan synthesis during cell growth, we hypothesized that loss of Rod complex activity during teichoic acid depletion would require increased peptidoglycan synthesis by another mechanism, and that this shift would cause changes in the abundance of specific types of peptidoglycan cross-links. To test this, we performed high-performance liquid chromatography (HPLC) on purified, lysozyme-digested peptidoglycan from exponentially growing wild-type and Δ*tagO* cells^49^. Deleting *tagO* caused a small increase in the percentage of uncross-linked peptidoglycan subunits, and a corresponding decrease in the percentage of dimeric cross-links (Fig. 1e and Extended Data Fig. 5a). Conversely, we observed increases in trimeric and tetrameric cross-links in Δ*tagO* cells that were reproducible but not significant across two replicates (Trimers: 9.9 ± 0.1% for Δ*tagO* vs 8.0 ± 0.7% for wild type; Tetramers: 1.6 ± 0.2% for Δ*tagO* vs 0.8 ± 0.1% for wild type. Error is s.e.m.; Extended Data Fig. 5b–d). The correlation of trimeric and tetrameric cross-linking with reduced Rod complex activity is consistent with a previous study that reported the reduction of these species in Δ*ponA* cells^49^.

### PBP1 activity is essential in the absence of teichoic acids

**Fig. 2:**
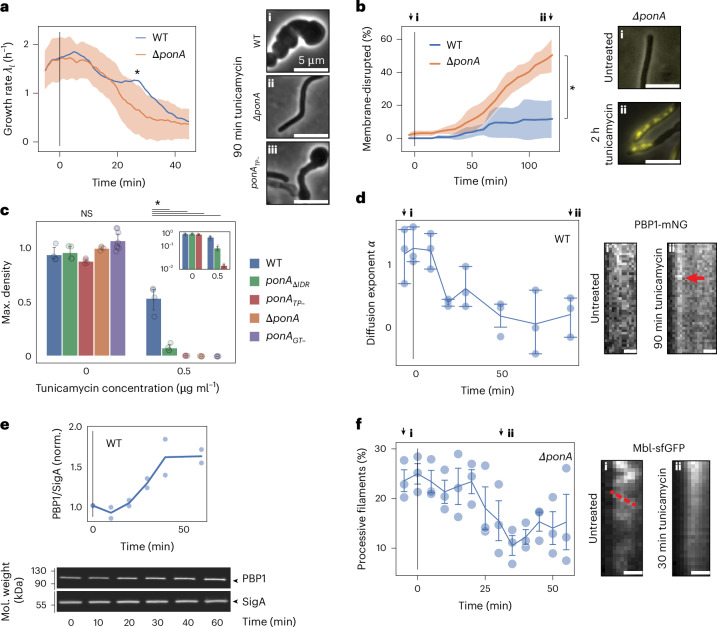
PBP1 is required for growth during wall teichoic acid synthesis inhibition. **a**, Left: cell length growth rate ${\lambda }_{l}=\frac{1}{l}\frac{{\rm{d}}l}{{\rm{d}}t}$ for Δ*ponA* cells during 0.5 *μg* *ml*^−1^ tunicamycin treatment. Data presented as smoothed median ± s.d. Blue line reproduces data from Fig. 1a. Significance calculated at 25 min using *t*wo-sided Student’s *t*-test, *p = 8 × 10^−33^. Right: micrographs showing (**i**) wild-type, (**ii**) Δ*ponA* and (**iii**) *ponA*_*TP−*_ cells at 90 min tunicamycin treatment. Scale bars, 5 μm. **b**, Left: percentage of membrane-disrupted cells following tunicamycin exposure. Plotted is the average ± s.d. across fields of view. Significance calculated for final timepoint using two-sided Student’s *t*-test, *p = 4 × 10^−6^. Right: phase-contrast micrographs overlaid with propidium iodide stain for *ΔponA* cells (**i**) before and (**ii**) after 2 h tunicamycin treatment. Scale bars, 5 μm. **c**, Maximum OD_600_ for wild-type, *ponA*_Δ*IDR*_*, ponA*_*TP−*_, Δ*ponA* and *ponA*_*GT−*_ cells ± tunicamycin. Plotted is the average across technical replicates (dots). Error bars show 95% confidence intervals. Inset: listed cell types plotted on log scale. Significance tested using one-way analysis of variance (ANOVA) for groups ± tunicamycin, followed by Tukey’s HSD post hoc test. Tunicamycin: *p = 5 × 10^−10^ (WT vs *ponA*_Δ*IDR*_), *p = 4 × 10^−11^ (WT vs *ponA*_*TP−*_), *p = 3 × 10^−11^ (WT vs Δ*ponA*), *p = 2 × 10^−12^ (WT vs *ponA*_*GT−*_); NS not significant. *ponA*_*GT−*_ was cultured with 30 mM xylose (inducer) + 30 mM MgCl_2_ (Methods). **d**, Left: diffusion exponent *α* of PBP1-mNeonGreen puncta throughout tunicamycin treatment (Methods). *α* = 1, diffusive; *α* < 1, subdiffusive. Error bars show 95% confidence intervals across replicates. Biological replicates shown as dots. Right: kymographs for (**i**) untreated and (**ii**) 90 min tunicamycin-treated cells. Arrow shows static PBP1-mNeonGreen localization. Total time, 4 s. Scale bars, 0.5 μm. **e**, Western blot of PBP1 levels during tunicamycin treatment. Top: PBP1 intensity, normalized by SigA and starting timepoint. Bottom: blot sections for PBP1-FLAG and identically loaded SigA control, processed in parallel. Biological replicates shown as dots. **f**, Left: percentage of processive Mbl-sfGFP filaments throughout tunicamycin treatment of Δ*ponA* cells. Data presented as average across biological replicates ±s.e.m. Biological replicates shown as dots. Right: representative fluorescent kymographs of Mbl motion across the cell waist in (**i**) LB and (**ii**) after 30 min tunicamycin treatment. Red dotted line follows a processive Mbl filament. Scale bars, 0.5 μm. See ‘Statistics and reproducibility’ in Methods for replicate counts.

**Fig. 3:**
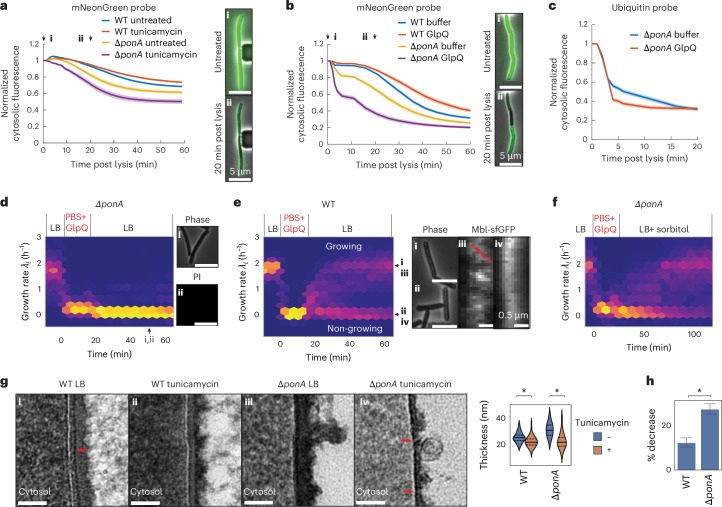
PBP1 fills pores that are exposed upon wall teichoic acid removal, conferring growth. **a**, Left: mNeonGreen diffusion out of membrane-disrupted cells following a 10-min (wild-type) or 20-min (Δ*ponA*) incubation with LB + tunicamycin or LB alone. Data plotted as average ± s.e.m. Right: micrographs showing fluorescence of tunicamycin-treated Δ*ponA* cells (**i**) before and (**ii**) 20 min after cell lysis. Scale bars, 5 μm. **b**, Left: same as **a** but for cells grown in LB then incubated in either PBS + 40 μM GlpQ in buffer A or PBS + buffer A before membrane disruption. Right: micrographs showing fluorescence of GlpQ-treated Δ*ponA* cells (**i**) before and (**ii**) 20 min after cell lysis. Scale bars, 5 μm. **c**, Ubiquitin-FlAsH diffusion out of membrane-disrupted Δ*ponA* cells following a 2-min incubation with PBS + 5% *N*-lauroylsarcosine + either 40 μM GlpQ or buffer A. Data plotted as average ± s.e.m. **d**, Left: heatmap of elongation rate ${\lambda }_{l}=\frac{1}{l}\frac{{dl}}{{dt}}$ vs time for Δ*ponA* cells following 15-min incubation with 10 μM GlpQ. Intensity shows cell counts binned by growth rate, normalized over time. Right: membrane integrity remains intact for Δ*ponA* cells 50 min after GlpQ incubation. (**i**) Phase contrast. (**ii**) Propidium iodide stain. Cf. Fig. 2b(ii). Scale bars, 5 μm. **e**, Left: same as **d** but for wild-type cells. Right: (**i**,**ii**) micrographs of (**i**) growing and (**ii**) non-growing cells; (**iii**,**iv**) kymographs of Mbl-sfGFP motion in (**iii**) growing and (**iv**) non-growing cells after GlpQ treatment. Scale bars, 5 μm in (**i**,**ii**), 0.5 μm in (**iii**,**iv**). Dashed red line in (**iii**) highlights a processive Mbl filament. **f**, Same as **d** but with exit from GlpQ coupled to hyperosmotic shock (500 mM sorbitol). **g**, Left: transmission electron micrographs of (**i**,**ii**) wild-type and (**iii**,**iv**) Δ*ponA* cell sidewalls (**i**,**iii**) before or (**ii**,**iv**) after 30 min tunicamycin treatment. Scale bars, 50 nm. Arrow in (**i**) shows electron-dense band, absent in tunicamycin-treated cells. Arrows in (**iv**) show cell wall thinning. Right: violin plots of cell envelope thickness. Dashed lines show quartiles, solid lines show median. **h**, Percentage decline in cell wall thickness for tunicamycin-treated cells relative to untreated cells (Methods). Data plotted as average ± s.e.m. See ‘Statistics and reproducibility’ in Methods for replicate counts and significance tests.

**Extended Data Fig. 6:**
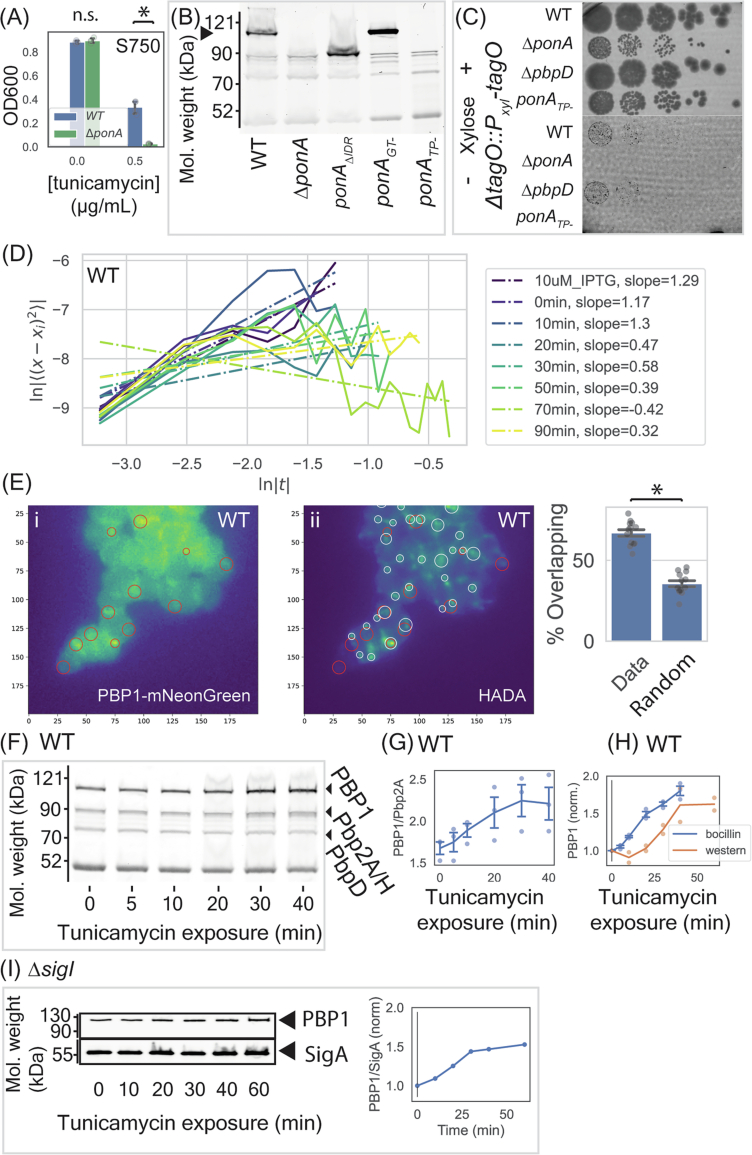
PBP1 activity and abundance increase and sustain growth during wall teichoic acid depletion. See *Methods: Statistics and Reproducibility* for replicate counts and significance tests. **(a)** Saturating OD600 for wild-type and Δ*ponA* cells ± tunicamycin when grown in S750 minimal media (*Methods*). Plotted is the average across technical replicates (dots) ± 95% confidence intervals. **(b)** SDS-PAGE on cell lysates from cultures incubated with fluorescently conjugated bocillin. Black arrow shows wild-type PBP1: present in wild-type and *ponA*_*GT−*_ lysates, shifted in *ponA*_Δ*IDR*_ lysates, absent in Δ*ponA* lysates, and not labeled in *ponA*_*TP−*_ lysates. **(c)** Spot assays for WT, Δ*ponA*, *ponA*_*TP−*_ and Δ*pbpD* mutant strains in a Δ*tagO P*_*xylA*_*-tagO* background, performed ± 30 mM xylose. **(d)** log-log plot of mean-squared displacement vs. time for PBP1 puncta during tunicamycin treatment. Slopes are diffusion exponents (*Methods*). **(e)** Co-localization of (i) PBP1-mNeonGreen expressed at a low level alongside the native copy of *ponA*, and (ii) HADA staining. 90 minutes tunicamycin exposure, 5 minutes HADA exposure. White circles show HADA puncta, red circles show PBP1 puncta. Inset: percentage of PBP1 puncta that overlap with HADA puncta when compared to a random distribution of PBP1 puncta. Data presented as average across 13 discrete fields of view (dots) ± SEM. **(f)** SDS-PAGE shows bocillin-PBP binding during tunicamycin treatment. Arrows show PBP1, PBP2A/PbpH/PBP2B (which could not be resolved by this method) and PbpD bands. Cultures treated with fluorescent bocillin for two minutes prior to cell lysis. **(g,h)** Ratio of PBP1 staining in (F) to **(g)** PBP2A/PBP2B and **(h)** PbpD. Values normalized to 0 min. PbpD staining showed no sustained trend during tunicamycin treatment. Bocillin data are presented as the average across biological replicates (dots) ± SEM. Western blot data reproduced from Fig. 2e. **(i)** Western blot analysis of PBP1 levels during tunicamycin treatment of a Δ*sigI* mutant. Left: Membrane staining for PBP1-FLAG and identically loaded SigA control, processed in parallel. Right: PBP1 staining relative to SigA, normalized to value at 0 min.

Since (1) Rod complexes and PBP1 are the two modes of peptidoglycan synthesis during cell growth, (2) teichoic acid depletion acutely inhibits Rod complex activity (Fig. 1c) and (3) cells lacking teichoic acids grow amorphously with a cell wall that is rich in trimeric and tetrameric cross-links, as would be expected from PBP1-mediated cell growth, we hypothesized that teichoic acid-less growth is executed by PBP1. To test this, we first measured the effect of tunicamycin treatment on the growth, shape and viability of Δ*ponA* mutants. At the single-cell level, tunicamycin caused a decrease in the growth rate of Δ*ponA* cells similar to the growth-rate decrease it caused in wild-type cells, except without the transient plateau and subsequent shape loss (Fig. 2a). Rather, Δ*ponA* mutants exhibited pervasive lysis after their growth rate decrease (Fig. 2b). Furthermore, concentrations of tunicamycin that permitted amorphous growth of wild-type cells prevented growth of Δ*ponA* cells (Fig. 2c and Extended Data Fig. 6a).

Teichoic acid-less cell growth was dependent on the glycosyltransferase activity of PBP1, since a mutant incapable of this reaction (*ponA*_*GT*_−) did not grow at concentrations of tunicamycin that permitted wild-type growth (Fig. 2c and Methods). Similarly, tunicamycin inhibited the growth of a PBP1 mutant incapable of transpeptidation (*ponA*_*TP−*_; Extended Data Fig. 6b)^50^ 40-times more strongly than wild-type growth (Fig. 2c), and caused only partial shape loss of this mutant before growth arrest (Fig. 2a, right). Tunicamycin may inhibit the growth of the *ponA*_*GT−*_ mutant more than it inhibits the growth of the *ponA*_*TP−*_ mutant because PBP1 transpeptidase activity is dependent on glycosyltransferase activity^51^. In addition to its catalytic domains, PBP1 also possesses an anionic 14.9 kDa C-terminal intrinsically disordered domain, which stimulates PBP1-mediated synthesis upon cell wall damage^26^; we found that tunicamycin inhibited the growth of a mutant harbouring a version of PBP1 lacking this domain (*ponA*_Δ*IDR*_; Fig. 2c and Extended Data Fig. 6b). Finally, transcriptional inhibition of *tagO* expression in Δ*ponA* and *ponA*_*TP−*_ mutant backgrounds prevented growth in bulk culture (Extended Data Fig. 6c).

These results demonstrate that PBP1 activity is essential for the amorphous cell growth following teichoic acid depletion, which inhibits Rod complexes. In this light, we hypothesized that amorphous growth would require increased peptidoglycan synthesis by PBP1 upon teichoic acid depletion, concomitant with the decline in Rod complex activity (Fig. 1c). To test this, we performed single-molecule tracking of a PBP1-mNeonGreen fusion protein expressed at low levels in addition to the wild-type protein at native levels (Fig. 2d, Extended Data Fig. 6d and Methods). Previous studies demonstrated that PBP1 is stationary when it is actively synthesizing peptidoglycan and rapidly diffuses when it is not^23,27^. Consistent with our hypothesis, we observed a stark decline in PBP1 diffusion upon tunicamycin treatment, culminating in large, stable PBP1 puncta (Fig. 2d, and Supplementary Videos 8 and 9).

To confirm that these PBP1 puncta were enzymatically active, we performed HADA staining after 90 min of tunicamycin treatment and measured the co-localization of PBP1 and HADA puncta. We found that 67% of PBP1 puncta overlapped with HADA puncta, while a simulated random localization yielded an overlap of only 36% (Extended Data Fig. 6e). Furthermore, Δ*ponA* cells treated with tunicamycin did not display HADA puncta (Fig. 1d), indicating that these puncta resulted from PBP1-polymerized peptidoglycan. However, *ponA*_*TP*−_ cells exhibited only a partial reduction in HADA puncta labelling compared to wild type (Fig. 1d), suggesting that other transpeptidases may partially complement PBP1 transpeptidation during teichoic acid depletion. This is consistent with the incomplete growth inhibition of tunicamycin-treated *ponA*_*TP−*_ cells, as opposed to the full arrest of tunicamycin-treated Δ*ponA* and *ponA*_*GT−*_ cells (Fig. 2c).

To test whether PBP1 expression increases in concert with PBP1-mediated synthesis upon teichoic acid depletion, we measured PBP1 abundance by western blot (Fig. 2e and Methods) and by labelling with the fluorescent penicillin bocillin, which covalently binds transpeptidases at their active sites^52^ (Extended Data Fig. 6f–h and Methods). Upon tunicamycin treatment, we observed a similar increase in PBP1 levels as measured with each assay (62 ± 8% by western blot, 80 ± 6% by bocillin labelling; errors are s.e.m.). Notably, the increase in PBP1 bocillin labelling preceded the increase in PBP1 staining by western blot (Extended Data Fig. 6h), on the same timescale as decreased PBP1 diffusion/synthesis. The increase in PBP1 expression did not result from the SigI stress response that is triggered by teichoic acid depletion^53^, since we also observed increased PBP1 expression in Δ*sigI* mutant cells (Extended Data Fig. 6i).

Our results demonstrate that teichoic acid depletion causes an increase in PBP1 abundance and PBP1-mediated synthesis, and that this enzyme becomes essential for growth without teichoic acids. Based on previous study^12^, the increase in PBP1 expression that we measured is, alone, insufficient to cause the degree of cell shape changes that we observed. Rather, our data are consistent with a model in which teichoic acids inhibit peptidoglycan synthesis by PBP1 such that when teichoic acids are depleted, PBP1 outcompetes Rod complexes for lipid II precursors, leading to slow, amorphous cell growth and Rod complex inhibition.

Given this model, we next questioned whether Rod complex motion also decreases in the absence of PBP1. We assayed Rod complex motion during tunicamycin treatment in Δ*ponA* cells (Methods), finding that Rod complex motion declined, but with a delay compared to wild-type cells (Fig. 2f, and Supplementary Videos 10 and 11). In this case, a mechanism besides competition for precursors must inhibit Rod complex function during teichoic acid depletion.

### Cell wall porosity increases without teichoic acids and PBP1

We next asked why PBP1 becomes activated during teichoic acid depletion, and why Rod complexes arrest during teichoic acid depletion in Δ*ponA* cells. Since PBP1 is thought to fill pores in the cell wall^23^ and is conditionally essential in the absence of teichoic acids, we hypothesized that teichoic acids play a complementary pore-filling role and that Rod complexes require a pore-less ‘confluent’ cell wall on which to synthesize new peptidoglycan. Furthermore, since intrinsically disordered domains sense cell wall damage^26^, probably through entropic translocation through pores in the cell wall^54^, and deleting PBP1’s intrinsically disordered region prevents growth during teichoic acid depletion (Fig. 2c), we additionally hypothesized that PBP1-mediated synthesis is stimulated by this domain when it senses pores in peptidoglycan that are exposed by teichoic acid depletion.

To test whether depleting teichoic acids results in pores within the cell wall, we leveraged a single-cell microfluidics assay we recently developed^55^. In this assay, we used a brief pulse of detergent to lyse cells expressing genetically encoded, cytosolic fluorescent probes and then measured the kinetics of the probes’ diffusion through the cell wall at the single-cell level, providing an empirical measurement of cell wall permeability (Methods). To measure the pore structure at different length scales, we used two probes: mNeonGreen (27.5 kDa, minimal radius *R*_min_ ≈ 2.0 nm^56^) and a fluorogenic version of ubiquitin that we developed (ubiquitin-FlAsH, 9.5 kDa, *R*_min_ ≈ 1.4 nm).

We first measured the effect of teichoic acid depletion on cell wall permeability by treating wild-type and Δ*ponA* cells with tunicamycin (Fig. 3a). To avoid confounding effects on cell wall permeability caused by changes in peptidoglycan synthesis, we measured permeability before Rod complex activity began to decline (10 min for WT and 20 min for Δ*ponA*). Contrary to our initial hypothesis, tunicamycin treatment of wild-type cells resulted in a modest but reproducible decrease in the permeability of wild-type cell walls, as measured with the mNeonGreen probe (Fig. 3a and Supplementary Fig. 2a). Conversely, the same treatment increased cell wall permeability in Δ*ponA* cells.

As an alternative approach to remove teichoic acids, we perfused cells with a purified phosphodiesterase, GlpQ, which *B. subtilis* normally expresses during phosphate starvation to digest and recycle its teichoic acids^57^ (Methods). Perfusing cells with GlpQ (dissolved in phosphate buffered saline, PBS) for 15 min reduced the abundance of teichoic acids within the cell wall (Supplementary Fig. 3 and Methods) and recapitulated the effect of tunicamycin on cell wall permeability: it caused a modest decrease in mNeonGreen permeability in wild-type cells but a large increase in permeability in Δ*ponA* cells (Fig. 3b and Supplementary Fig. 2b). Together, these results are consistent with a model in which teichoic acids occlude pores in the cell wall, and in which PBP1 is activated by these pores via its intrinsically disordered domain to ‘seal’ them, thereby preventing an increase in cell wall permeability^55^.

**Extended Data Fig. 7:**
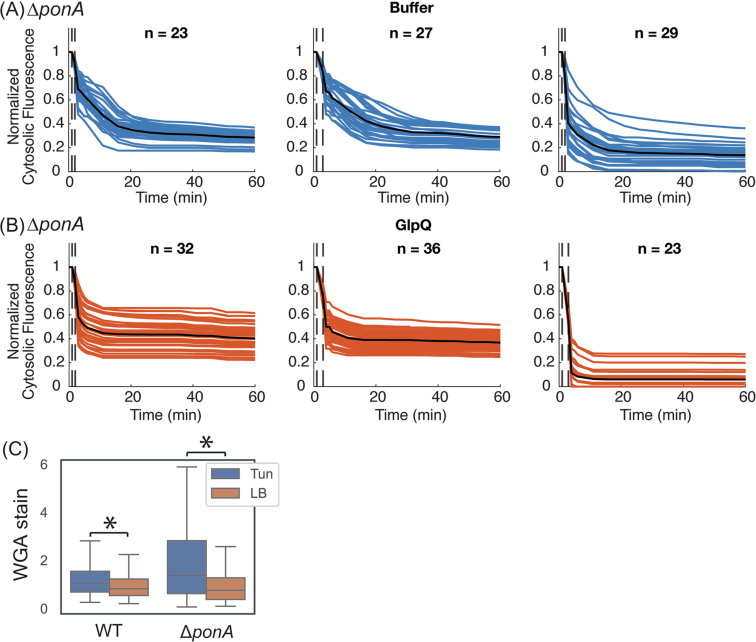
Ubiquitin-FlAsH immediately leaves the cytosol upon wall teichoic acid digestion and simultaneous lysis in Δ*ponA* cells. **(a-b)** Individual Ubiquitin-FlAsH fluorescent traces and biological replicates following 2 min incubation with PBS + 5% N-lauroylsarcosine and either **(a)** Buffer A alone, or **(b)** 40uM GlpQ. Data plotted from 79 cells (Buffer A) and 91 cells (GlpQ). Three biological replicates per condition. **(c)** Box plots showing envelope fluorescence of cells labeled with a wheat-germ agglutinin-AlexaFluor488 conjugate after incubation with LB alone or LB supplemented with tunicamycin for either 10 min (wild-type) or 20 min (Δ*ponA*). Box lines show quartiles, whiskers show spread of data (excluding outliers for ease of presentation). n = 538 cells (wild-type, LB), n = 271 cells (wild-type, tunicamycin), n = 285 cells (Δ*ponA*, LB), n = 288 cells (Δ*ponA*, tunicamycin). 3 biological replicates per condition. Significance tested by two-sided Student’s *t*-test, *p* = 3e-7 (WT LB v. WT tunicamycin), *p* = 2e-12 (Δ*ponA* LB v. Δ*ponA* tunicamycin).

Treating Δ*ponA* cells with tunicamycin or GlpQ for durations approaching the cell-cycle time (~20 min) could cause pore formation indirectly, for example, by inducing peptidoglycan hydrolysis unbalanced by synthesis^34–37^. To control for this possibility, we measured cell-wall permeability during teichoic acid digestion by GlpQ, reasoning that at short time scales after teichoic acid digestion, peptidoglycan hydrolases would not have time to meaningfully enlarge pores. Because we anticipated small incipient pores, we used our ubiquitin probe to test for them. When we simultaneously digested teichoic acids and measured the loss of this probe in Δ*ponA* cells, we observed an increase in cell wall permeability within 2 min of GlpQ treatment (Fig. 3c, Extended Data Fig. 7a,b and Supplementary Fig. 2c).

In concurrent work, we discovered that the size threshold for cell wall permeability is just below the size of typical globular proteins^55^. We therefore hypothesized that the pores exposed during teichoic acid depletion would cause increased labelling by peptidoglycan-binding proteins. To test this, we measured whether teichoic acid depletion affected cell-wall labelling with a fluorescent lectin that specifically binds to the *N*-acetyl glucosamine moiety of peptidoglycan. We observed an increase in labelling in both Δ*ponA* cells and, to a lesser extent, wild-type cells following pre-treatment with tunicamycin (Extended Data Fig. 7c). We speculate that teichoic acid depletion in wild-type cells reduces cell wall permeability (Fig. 3a) but increases lectin binding because PBP1 is membrane bound and would therefore only fill pores near the internal surface of the cell wall, leaving teichoic acid-less peptidoglycan on the outer surface exposed.

**Extended Data Fig. 8:**
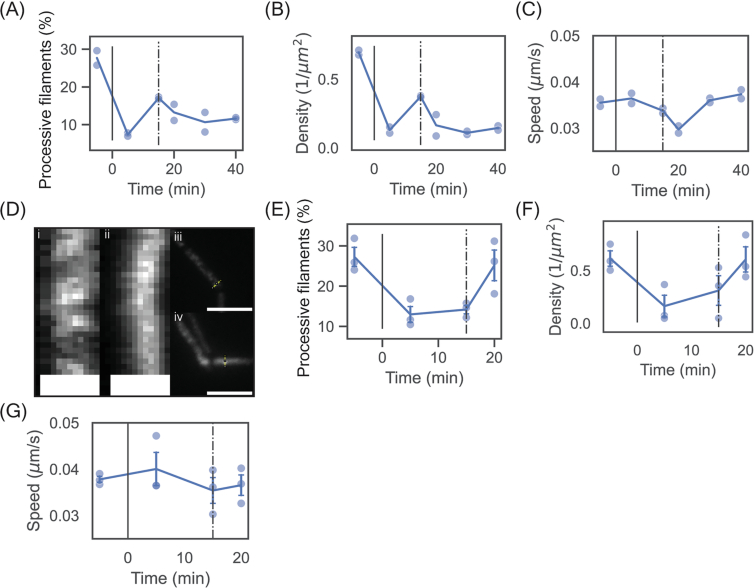
Processive Rod complex motion recovery is limited to growing cells following GlpQ treatment. **(a-c)** Mbl-sfGFP activity time course during 25 μM GlpQ treatment, which limits the growth recovery of wild-type cells. Data are presented as average across biological replicates. Analysis performed on 10,976 discrete filament tracks from 2 biological replicates. Solid vertical line shows onset of GlpQ treatment. Dashed vertical line shows release from GlpQ. **(a)** Percentage of processive filaments. **(b)** Spatial density of processive Mbl-sfGFP filaments. **(c)** Speed of processive Mbl-sfGFP filaments. **(d)** Representative kymographs from TIRF imaging of (i) growing and (ii) non-growing wild-type cells 35 min after release from GlpQ treatment. Scale bars 1 μm. (iii-iv) TIRF micrographs showing whole cells during a single timepoint of kymographs in (i-ii) respectively. Yellow lines show kymograph traces. Scale bars 5 μm. **(e-g)** Mbl-sfGFP and MreB-mNeonGreen activity time course during PBS treatment. Data are presented as average across biological replicates ± SEM. Analysis performed on 8,424 discrete filament tracks from 3 independent experiments. Data pooled from experiments with 15 min and 20 min PBS exposure. Solid vertical line shows onset of PBS treatment. Dashed vertical line shows release from PBS. **(e)** Percentage of processive filaments. **(f)** Spatial density of processive Mbl-sfGFP filaments. **(g)** Speed of processive filaments.

Our model that Rod complexes require a pore-less cell wall as their substrate made the prediction that removing teichoic acids from Δ*ponA* cells by perfusing them with PBS-suspended GlpQ (which arrests cell growth and leads to a permeable cell wall) would prevent Rod complex activity and cell growth upon re-immersion in growth media. Consistent with this prediction, Δ*ponA* cells were unable to recover from teichoic acid digestion but did not lyse upon re-immersion in growth media (Fig. 3d), whereas most wild-type cells recovered (Δ*ponA*: 7 ± 2% recovered; wild-type: 66 ± 2% recovered; recovery counted as reaching half the initial unperturbed growth rate; error is s.e.m.; Fig. 3e). Furthermore, wild-type cells that resumed growth did so as rods and recovered Rod complex activity (Fig. 3e(i,iii)), while cells that did not recover showed no Rod complex motion (Fig. 3e(ii,iv) and Extended Data Fig. 8a–d). Rod complex activity and cell growth of both wild-type and Δ*ponA* cells recovered fully from incubation in PBS alone (wild-type: 94 ± 1% recovered growth; Δ*ponA*: 81 ± 2% recovered growth; error is s.e.m.; Extended Data Fig. 8e–g and Supplementary Fig. 4a,b). This correlation between pores, Rod complex activity and PBP1 is strong evidence that the Rod complexes cannot add peptidoglycan to a porous cell wall. However, since GlpQ treatment of wild-type cells does not induce pores (Fig. 3c) but partially inhibits growth recovery (Fig. 3e), teichoic acid digestion must additionally inhibit Rod complexes via a mechanism independent of pores, at least of those that are detected by the mNeonGreen probe. We speculate that this results from the effect of spatially heterogeneous peptidoglycan synthesis by PBP1 on Rod complexes (Discussion).

We reasoned that if pores in the cell wall inhibit Rod complex activity upon teichoic acid removal, then shrinking these pores would activate Rod complexes, thereby promoting cell growth recovery after GlpQ treatment. We tested this prediction by coupling the re-immersion of cells in rich media following GlpQ treatment to a 500-mM hyperosmotic shock that physically contracted the cell wall^58^. To ensure that any cell growth recovery resulted directly from cell wall contraction and not from PBP1 activity, we performed this experiment in Δ*ponA* cells that are otherwise unable to grow following teichoic acid cleavage. Consistent with our hypothesis, we found that hyperosmotic shock led to a partial growth recovery after teichoic acid removal even in the absence of PBP1 (60 ± 2% recovered; error is s.e.m.; Fig. 3f and Supplementary Fig. 4c).

**Extended Data Fig. 9:**
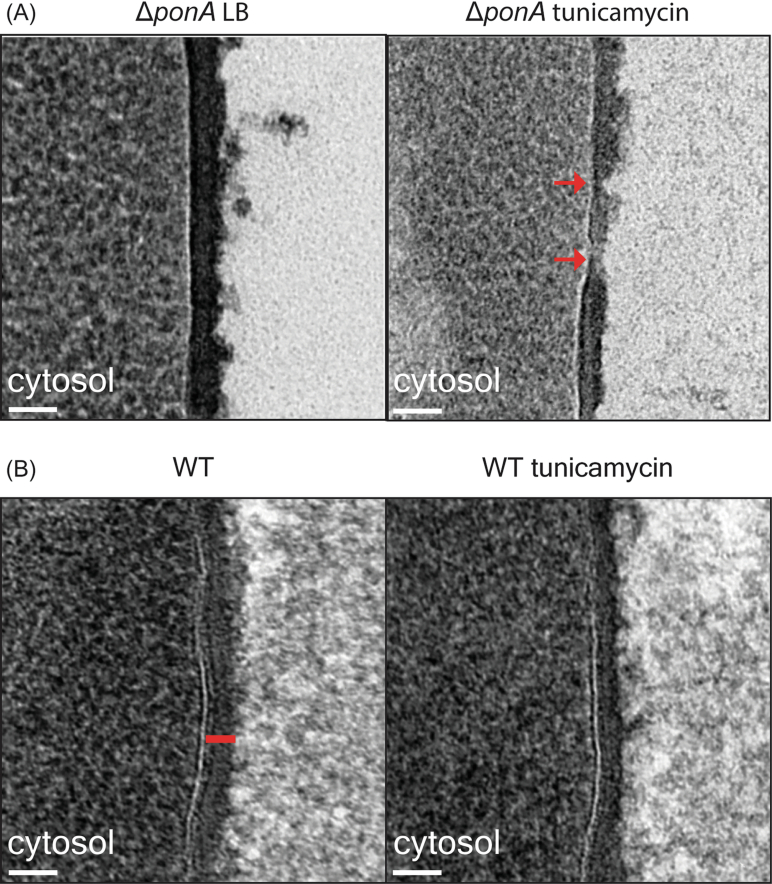
Cell wall degradation occurs during wall teichoic acid depletion in Δ*ponA* cells. **(a-b)** Transmission electron micrographs of the sidewall of exponentially growing cells grown in either LB or LB supplemented with 0.5 μg/mL tunicamycin for 30 min. Scale bars 50 nm. Representative images taken from 47 cells (Δ*ponA* LB), 44 cells (Δ*ponA* tunicamycin), 31 cells (wild-type LB) and 28 cells (wild-type tunicamycin). One biological replicate. **(a)** Δ*ponA* cells. Red arrows show sites of severe cell wall thinning during tunicamycin treatment. **(b)** Wild-type cells. Red bar shows the region used to calculate cell wall thickness, measured from the edge of the cell membrane (white line).

Finally, we sought to visualize the effect of teichoic acid depletion on the cell wall. Previously, atomic force microscopy explicitly aimed at resolving peptidoglycan structure did not resolve a difference between isolated cell wall sacculi before and after hydrofluoric acid treatment to remove teichoic acids^59^. Therefore, we used transmission electron microscopy (TEM) to examine the effect of teichoic acid depletion on cell wall ultrastructure. To do this, we performed high-pressure freeze-substitution staining on chemically fixed wild-type and Δ*ponA* cells that had been grown either in LB alone or with tunicamycin treatment for 30 min (Methods). We observed significant cell wall degradation of Δ*ponA* tunicamycin-treated cell walls, visible both as general thinning of the cell wall relative to untreated cells and as localized regions of lower density within the wall (Fig. 3g and Extended Data Fig. 9a). Wild-type cells showed a smaller relative decline in cell wall thickness during tunicamycin treatment (Fig. 3g,h and Extended Data Fig. 9b). This small decline in wild-type cells is consistent with previous observations of a teichoic acid-dependent layer supporting the Gram-positive periplasm in *Streptococcus pneumoniae*^60^, visible here as an electron-dense band on the inner face of the untreated cell wall that is absent in tunicamycin-treated cells (Fig. 3g). Although this technique is incapable of resolving the nanoscale pores detected by our permeability assays, these findings are consistent with the conclusions that (1) PBP1 fills pores within the cell wall during teichoic acid depletion and (2) in Δ*ponA* cells, teichoic acid depletion causes significant cell wall degradation, leading to a loss of Rod complex activity.

### *lytE* becomes essential during teichoic acid depletion

**Extended Data Fig. 10:**
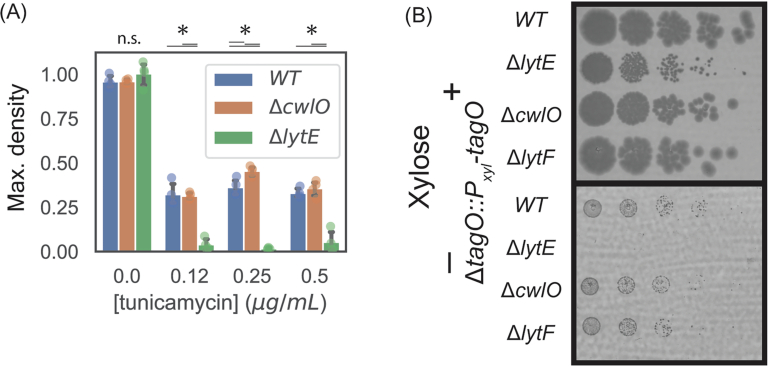
*lytE* is required for growth in LB medium following wall teichoic acid depletion. **(a)** Saturating culture density in various concentrations of tunicamycin for wild-type, Δ*cwlO* and Δ*lytE* cells grown in LB medium. Plotted is the average across 4 technical replicates (individual replicates shown as dots) for one representative biological replicate out of two. Error bars show 95% confidence intervals based on bootstrap analysis. Statistical significance tested with one-way ANOVA followed by Tukey’s HSD post-hoc test for different concentrations of tunicamycin. 0.125 μg/mL tunicamycin: *p* = 2e-5 (WT v. Δ*lytE*), *p* = 3e-5 (Δ*cwlO* v. Δ*lytE*). 0.25 μg/mL tunicamycin: *p* = 8e-3 (WT v. Δ*cwlO*), *p* = 3e-7 (WT v. Δ*lytE*), *p* = 4e-8 (Δ*cwlO* v. Δ*lytE*). 0.5 μg/mL tunicamycin: *p* = 4e-5 (WT v. Δ*lytE*), *p* = 2e-5 (Δ*cwlO* v. Δ*lytE*). **(b)** Spot assays showing growth with and without *tagO* transcriptional inhibition in *P*_*xylA*_*-tagO* cells with the mutant backgrounds shown (two biological replicates).

Finally, we questioned whether the cell wall degradation that occurs upon teichoic acid depletion in Δ*ponA* cells (Fig. 3a,b,g and Extended Data Fig. 9a) depends on specific peptidoglycan hydrolases. Although *Bacillus subtilis* encodes up to 42 putative hydrolases, cell growth can be sustained by either of two synthetically lethal D,L endopeptidases: CwlO or LytE^61^. Furthermore, teichoic acid depletion significantly increases SigI-dependent *lytE* expression^53^. Therefore, we measured the growth rate and width dynamics of Δ*cwlO* and Δ*lytE* cells upon tunicamycin treatment in the presence or absence of PBP1, and found that the initial phases of these dynamics were identical to those of the respective background strains possessing both hydrolases (Supplementary Fig. 5a–d). In addition, upon tunicamycin treatment, Rod complex activity decreased in both hydrolase mutants, as it did in wild-type cells (Supplementary Fig. 5e–j). Strikingly, at long times after either tunicamycin treatment (Extended Data Fig. 10a), or transcriptional inhibition of *tagO* (Extended Data Fig. 10b), Δ*lytE* cells failed to grow. This means that *lytE*, similar to *ponA*, is essential during teichoic acid depletion.

## Discussion

**Fig. 4:**
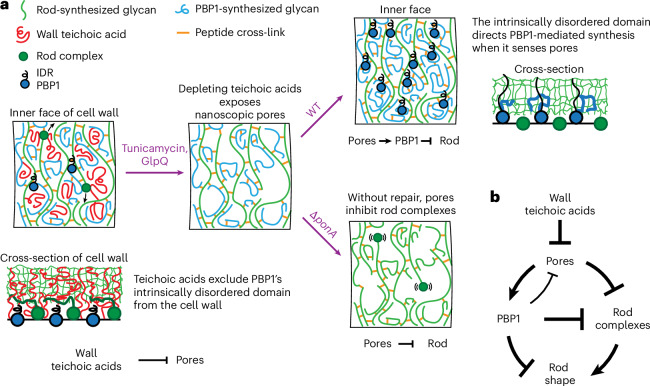
Model: wall teichoic acids maintain rod-shape by occluding nanoscale pores. **a**, Model illustration. During teichoic acid depletion of wild-type cells, denuded cell wall pores are targeted by PBP1, competitively inhibiting Rod complexes and driving localized peptidoglycan synthesis. This isotropic cell wall synthesis causes decreased growth and loss of cell shape. During teichoic acid depletion of Δ*ponA* cells, denuded cell wall pores directly arrest Rod complex motion through depletion of cross-linking acceptors. **b**, Schematic of model described in **a**.

Our data support a model in which teichoic acids occlude nanoscopic pores in peptidoglycan (Fig. 4a,b) and prevent them from growing, which promotes rod shape by inhibiting PBP1 and enabling Rod complex activity. According to this model, in wild-type cells, teichoic acids limit PBP1’s access to natural pores within peptidoglycan by ‘filling’ them and/or by ‘lining’ the inner face of the cell wall (Fig. 4a). When teichoic acids are depleted, PBP1’s intrinsically disordered domain rapidly senses the pores, leading to an increase in the relative amount of peptidoglycan that PBP1 synthesizes (Fig. 2d); we find it likely that the anionic teichoic acids exclude the anionic intrinsically disordered domain from pores in the cell wall through polymer–polymer repulsion, similar to the forces that govern macromolecular crowding^62^. Teichoic acid depletion also leads to an increase in PBP1 abundance (Extended Data Fig. 6f–i) via an unknown mechanism. PBP1 is therefore the essential peptidoglycan synthase driving amorphous growth. Notably, our model explains previous observations that Rod complexes become non-essential during teichoic acid depletion^63^.

At slightly longer times, teichoic acid depletion leads to decreased activity of Rod complexes (Fig. 1c). According to our model, this occurs in wild-type cells because increased PBP1-mediated synthesis inhibits Rod complex activity by reducing the lipid II pool. Reducing lipid II synthesis is known to inhibit Rod complex activity in both fast and slow growth conditions^21^, and we observed a reduction in Rod complex activity during teichoic acid depletion in both conditions (Fig. 1c and Extended Data Fig. 3g–i). We speculate, however, that the heterogeneous spatial distribution of PBP1-synthesized peptidoglycan upon teichoic acid depletion amplifies the inhibition of Rod complexes because one function of PBP1 in untreated cells is to contribute to a dense, homogeneous ‘lawn’ of acceptor peptides. This hypothesis is consistent with the impaired Rod complex function of Δ*ponA* mutants during vegetative growth^64^, which we suppressed with supplemental magnesium (Methods). Localized PBP1 synthesis could inhibit Rod complexes if the latter could only cross-link efficiently to recently synthesized peptidoglycan (or peptidoglycan that is still being synthesized) due to either spatial or biochemical maturation of acceptor peptides (for example, proximity to the plasma membrane). The heterogeneity of immature peptidoglycan would not inhibit PBP1 since it is not spatially processive, and can therefore cross-link to its own products.

In the absence of PBP1, teichoic acid depletion causes the simultaneous formation of nanoscopic pores in the cell wall (Fig. 3c) and the eventual arrest of Rod complexes (Figs. 2d and 3d). According to our model, the pores exposed by teichoic acid depletion enlarge through hydrolysis without repair by PBP1 (Figs. 3a,b and 4a). When the pores become too large, the density of peptide acceptors becomes so low that Rod complexes arrest for the same reason that pharmacological inhibition of Class B PBP transpeptidation arrests Rod complexes. Conversely, when these pores are contracted, Rod complex activity can recover (Fig. 3f). The delay in Rod complex arrest that we observed in Δ*ponA* mutants compared to that in wild-type cells is consistent with competitive inhibition of Rod complexes by PBP1.

In addition to cell wall synthesis, teichoic acids also influence peptidoglycan hydrolysis during cell growth, both directly and indirectly^34–37,53^. We found that *lytE* becomes essential during teichoic acid depletion for cells grown in LB, meaning that stable amorphous teichoic acid-less growth is driven by PBP1 and LytE. However, deletion of *cwlO* or *lytE* had no effect on the growth, shape and Rod complex dynamics at early times after teichoic acid depletion, indicating that teichoic acids do not differentially regulate these two enzymes. Whether this conditional essentiality of *lytE* arises due to CwlO becoming inactive at long times following teichoic acid depletion will be the subject of further study. Teichoic acids’ impact on hydrolysis could also underlie our observation that GlpQ-mediated digestion of teichoic acids partially prevents recovery of wild-type cells even though PBP1 activity prevents the formation of large pores (Fig. 3e). For example, runaway PBP1 activity and hydrolysis during GlpQ treatment could create an irreversibly altered cell wall incapable of supporting Rod complex activity.

*B. subtilis* cells depleted for wall teichoic acids often fail to separate and show aberrantly thick septa^10,19^. Our data are consistent with increased PBP1-mediated synthesis contributing to septal peptidoglycan synthesis since Δ*ponA* cells show reduced septal HADA labelling relative to wild-type cells during tunicamycin treatment (Fig. 1d). However, since teichoic acid-depleted cells that are forced to maintain rod shape through confinement grow and divide faster than their spherical counterparts^19^, the loss of rod shape clearly contributes substantially to this division defect.

In sum, wall teichoic acids are critical regulators of rod-shaped morphogenesis in *Bacillus subtilis* since they coordinately influence both modes of peptidoglycan synthesis as well as its hydrolysis. Our study resolves the longstanding question of how teichoic acids promote rod shape without directly contributing to cell wall anisotropy. Furthermore, we established a direct link between the molecular structure of the cell wall and anisotropic cell wall synthesis, identifying the cell wall as a critical autoregulatory factor of its own synthesis, rather than a passive substrate.

## Methods

### Microscopy

All timelapse microscopy was performed with a Nikon Eclipse Ti2 microscope, outfitted with a photometrics PRIME BSI CMOS camera (Teledyne Photometrics) and a Nikon ×100 Plan Apo Phase Contrast objective, NA 1.45. All non-TIRF (total internal reflection fluorescence) imaging used LED illumination. We conducted all imaging using CellASIC ONIX microfluidic plates (Millipore, B04A-03), using standard priming and media exchange protocols. We heated all microscope stage components, CellASIC plates and objectives to 37 °C before imaging using a microscope incubator outfitted with a heater. We performed our timelapse phase-contrast microscopy using 20-s timesteps, monitoring medium exchange using the dye AlexaFluor 647 at a final media concentration of 2.5 μg ml^−1^, with CY5 illumination adjusted to avoid any confounding effects of phototoxicity.

### TIRF microscopy

All TIRF microscopy was performed with the same Nikon Eclipse Ti2 microscope, using a COHERENT 488 nm laser and ×100 Plan Apo objective, NA 1.45, with 2-s timesteps. We adjusted our laser power, exposure time and TIRF alignment to minimize photobleaching, and used a fresh field of view for each new timepoint to avoid any confounding effects of phototoxicity.

### Single-molecule tracking

We performed single-molecule tracking by imaging small fields of view with TIRF microscopy, using a timestep of 90 ms, 1% laser power and 40 ms camera exposure time. To facilitate rapid image acquisition, the laser constantly remained on for the 4-s acquisition. To facilitate single-molecule resolution, we induced PBP1-mNeonGreen expression at low levels from a heterologous HyperSpank promoter with 10 μM isopropyl *ß*-D-1-thiogalactopyranoside (IPTG), alongside unperturbed native PBP1 expression from the endogenous locus. Of note, Supplementary Videos 8 and 9 were acquired with imaging conditions that visually emphasize the duration of PBP1 puncta during teichoic acid depletion, but that were not used for single-molecule tracking because of their low time-resolution: time step = 500 ms, exposure time 50 ms, 15 s timecourse, 1% laser power. We generated our image file structures for downstream analysis using the ImageJ script ‘tirf_pbp1_processing_v1.ijm’. We then performed spot tracking using the Python script ‘single_molecule_tracking_v1.py’, which detected and tracked filaments as follows: we detected puncta with SciKitImage’s bloblog feature using manually curated thresholds to start each timelapse (which were then preserved within all timelapses acquired using that imaging condition). These thresholds were iteratively reduced throughout each step of a single timecourse to maintain roughly equivalent levels of spot detection. This allowed us to correct for partial photobleaching of the fluorescent signal. We tracked individual puncta across successive images (all analysed images used a timestep of 90 ms) by calculating the minimum distance between all annotated puncta, subject to a maximum displacement threshold of two pixels (equivalent to a linear distance of 186 nm based on our magnification and camera size). We then filtered to exclude all tracks with length of 2 timepoints or less. Finally, we inferred the diffusion exponents by applying linear regression to log–log plots for the population-averaged mean-squared displacement of individual PBP1 puncta over time.

### Cell culture and transformation

Unless otherwise stated, all cell culture was performed with LB media (Luria Broth, Lennox). All in vivo imaging and spot-assay experiments were performed with log-phase cultures of optical density at 600 nm (OD_600_) ≤ 0.2. Transformations were performed with competent *B. subtilis* cells grown in MC media + 20 mM MgSO_4_, then plated onto LB agarose plates. All cells were congenic with a PY79 strain background. Strains are tabulated in Supplementary Table 1. We note that expression of *ponA*_*GT−*_*(E115A)* at high levels was toxic to cells and required media supplementation with 30 mM MgCl_2_. Similarly, our experiments on Rod complex tracking in Δ*ponA* cells were performed with 10 mM supplemental MgCl_2_ to allow visualization of processive Rod complexes in this strain background.

### S750 media

We used S750 defined minimal media following a recipe used previously^65^: 1× S750 salts, 1× S750 metals, 1% glucose, 0.1% glutamate + deionized water (dH_2_O) to final volume.

10× S750 salts: 0.5 M 3-(N-morpholino)propanesulfonic acid (adjusted to pH 7.0 with KOH), 100 mM ammonium sulfate, 50 mM potassium phosphate monobasic (adjusted to pH 7.0), filter sterilized.

100× S750 metals: 0.2 M MgCl_2_, 70 mM CaCl_2_, 5 mM MnCl_2_, 0.1 mM ZnCl_2_, 100 μg ml^−1^ thiamine HCl, 2 mM HCl, 0.5 mM FeCl_3_, dH_2_O to final volume.

We performed our S750 experiments by first inoculating overnight S750 cultures from LB plates, then back diluting the following morning into fresh S750 media and allowing these new cultures to grow to exponential phase before starting an experiment.

### Click labelling of peptidoglycan precursors

We treated exponentially growing cultures with 1 mM EDA-DA (Fisher Scientific, 771410), which is incorporated into peptidoglycan precursors cytosolically^48^, for 20 min at 37 °C. We then washed cell cultures once in PBS before fixing cells for 20 min on ice in PBS supplemented with 3.6% paraformaldehyde. We labelled the EDA-DA alkyne group with Alexa Fluor 488 Azide (A10266) by following the Invitrogen Click-iT Cell Reaction Buffer kit (Thermo Fisher, C10269), but without supplementing with BSA (to ensure optimal diffusion through the cell wall). We spotted cells onto PBS pads made with 2% agarose and imaged all samples within 18 h of click labelling. We generated different treatment conditions by inoculating identical prewarmed tubes of fresh growth media from a single exponentially growing culture. We generated our tunicamycin-treated samples by pretreating for 10 min with 0.5 μg ml^−1^ tunicamycin before EDA-DA addition, for a total of 30 min drug exposure. By contrast, we added 50 μg ml^−1^ fosfomycin or 1 μg ml^−1^ vancomycin simultaneous with EDA-DA for a total of 20 min drug exposure. Staining decreased only partially for vancomycin treatment relative to LB alone, consistent with AF488 labelling both nascent peptidoglycan and lipid II.

### Protein purification and application

Protein was purified according to the following previously published method^57^: We incubated cell cultures of bER461 in 500 ml LB + 100 μg ml^−1^ ampicillin and 35 μg ml^−1^ chloramphenicol at 37 °C with shaking until they reached an OD_600_ of 0.6–0.8. We then shifted these cultures to 16 °C, added IPTG to a final concentration of 0.5 mM and incubated them with shaking for 18 h. We centrifuged these cultures at 3,720*g* for 10 min at 4 °C, resuspended them in buffer A (50 mM Tris-HCl + 200 mM NaCl + 1 mM CaCl_2_ + 1 mM MgCl_2_, pH 8.0) + 25 mM imidazole and lysed them by disruption in an Emulsiflex-C5. After disruption, we incubated the supernatant with a column containing HisPur Ni-NTA Resin (Thermo Fisher, 88221), ran this column over an imidazole gradient and visualized samples via SDS–PAGE with stain-free labelling (Bio-Rad, 1610183). We pooled column elutions containing high protein concentrations, performed dialysis at 4 °C overnight in buffer A, concentrated our samples with Amicon Ultra Centrifugal Filters (10 kDa molecular weight cut-off), and flash froze single-use aliquots with liquid nitrogen for storage at −80 °C. We calculated our protein concentration using a Nanodrop One Micro-UV/Vis spectrophotometer (ThermoFisher).

The Gram-positive cell wall imposes a substantial diffusion barrier to molecules above 15 kDa^55^. At 30.1 kDa, GlpQ exceeds this limit substantially, rendering it necessary to partially degrade the cell wall to allow GlpQ access to its target substrates. We accomplished this by performing all GlpQ incubations in PBS lacking divalent cations for the times listed. This incubation simultaneously arrested peptidoglycan synthesis and induced autolysis (cell wall degradation) by the cells’ native autolysins. The PBS used to perform these incubations was prepared according to the following recipe^66^ (10× PBS, 1 l): 80 g NaCl, 2 g KCl, 14.4 g Na_2_HPO_4_, 2.4 g KH_2_PO_4_, with pH adjusted to 7.4 using HCl.

### In vivo bocillin labelling

We grew cell cultures to early log-phase, back diluted them into prewarmed LB to create multiple identical log-phase samples, and added tunicamycin (MP Biomedical, 0215002805; Cell Signaling Technology, 12819) to each sample at successive time intervals of 10 min to a final concentration of 0.5 μg ml^−1^. Before lysis, we treated all cultures with bocillin-FL (Invitrogen, B13233) simultaneously to a final concentration of 1 μg ml^−1^, incubated them for 2 min, then pelleted cells for cell lysate extraction. We treated cell pellets with lysis buffer (20 mM Tris pH 7.5, 1 mM EDTA, 10 mM MgCl_2_, 1 mg ml^−1^ lysozyme, 1 mM PMSF, 10 μg ml^−1^ DNAse I, 100 μg ml^−1^ RNAse A). We added fresh lysozyme, PMSF, DNAse I and RNAse A to a lysis buffer stock for each preparation. After lysis, we added sample buffer (Invitrogen, LC2570). We then visualized cell lysates via SDS–PAGE in running buffer (25 mM Tris, 192 mM glycine, 0.1% SDS) with 10% Acrylamide FastCast gels (Bio-Rad, 1610183) using a Cytiva Amersham Typhoon fluorescent imager. We used an iBright prestained protein ladder (Invitrogen, LC5615) and normalized loading volumes on the basis of bulk culture OD_600_ readings.

### Western blotting

We grew cell cultures to early log-phase, back diluted them into prewarmed LB to create multiple identical log-phase samples, and added tunicamycin (MP Biomedical, 0215002805; Cell Signaling Technology, 12819) to each sample at successive time intervals of 10 min to a final concentration of 0.5 μg ml^−1^. We then pelleted cells for cell lysate extraction and treated cell pellets with lysis buffer (20 mM Tris pH 7.5, 1 mM EDTA, 10 mM MgCl_2_, 1 mg ml^−1^ lysozyme, 1 mM PMSF, 10 μg ml^−1^ DNAse I, 100 μg ml^−1^ RNAse A). We added fresh lysozyme, PMSF, DNAse I and RNAse A to a lysis buffer stock for each preparation. After lysis, we added sample buffer (Invitrogen, LC2570) and then performed SDS–PAGE in running buffer (25 mM Tris, 192 mM glycine, 0.1% SDS) with 10% Acrylamide FastCast gels (Bio-Rad, 1610183) with two identically loaded replicates to act as a loading control. We normalized loading volumes on the basis of bulk culture OD_600_ readings and used an iBright prestained protein ladder (Invitrogen, LC5615). After SDS–PAGE, we transferred products to PVDF membranes (Thermo Scientific, 22860) in transfer buffer (25 mM Tris, 192 mM glycine, 20% methanol), followed by washing using deionized water and blocking with blocking buffer (Thermo Scientific, 37565). We performed our primary antibody labelling separately for each replicate using either a monoclonal IgG rabbit anti-FLAG antibody (2,500× dilution, Genscript, A01868-40) or a rabbit IgG anti-SigA antibody (10,000× dilution, gift from the laboratory of Masaya Fujita)^67^. We performed secondary antibody labelling with goat anti-rabbit IgG AlexaFluor 647 (2,500× dilution, Invitrogen, A-21245), and visualized our blots using a Cytiva Amersham Typhoon fluorescent imager. We consistently observed off-target labelling with our anti-FLAG-treated blots, so we confirmed the identity of the *ponA*-FLAG band using a strain lacking this marker.

### Wall teichoic acid measurement by flow cytometry

We grew cell cultures to log-phase (OD_600_ = 0.1–0.2), then took two samples from a single cell culture, washed samples once in PBS and incubated them in PBS + 40 μM GlpQ or PBS + the equivalent volume of buffer A for 15 min with rocking at 37 °C. We then washed cells again once in PBS and resuspended them in PBS supplemented with 200 μg ml^−1^ of Concanavalin A-AlexaFluor 647 (Invitrogen, C21421) for 10 min at 37 °C with rocking. Concanavalin A is a lectin that binds glycosylation modifications along the teichoic acid polymer^41,68^. We washed cells again once in PBS, then resuspended samples in PBS for flow cytometry. We performed our flow cytometry measurements with a Cytek Aurora spectral flow cytometer (Cytek Biosciences) set to 10,000 events per sample, with gains adjusted to capture the full dynamic range of our population measurements. We consistently calibrated the instrument to a low background (<10 events per second) and vigorously vortexed each sample before acquisition. Since we were interested in statistics of the whole cell population, we performed minimal gating (Extended Data Fig. 6b). Since GlpQ preferentially cleaves non-glycosylated teichoic acids^57^, the GlpQ-mediated reduction in Concanavalin A staining for wild-type cells is probably an underestimate of GlpQ-mediated teichoic acid cleavage. We used Δ*tagE* mutants to control for non-specific wheat-germ agglutinin labelling since TagE is responsible for teichoic acid glycosylation^69^.

### Image analysis and tracking

Cell tracking was performed using custom scripts written in FIJI (imagej.net), MATLAB (Mathworks) and Python, all of which are available online at https://github.com/felixbarber/WTA_peptidoglycan_nanostructure. We first applied the ImageJ scripts ‘image_process_updated.ijm’ and ‘alignment_updated.ijm’ (for timelapse imaging, for example, Fig. 1b) or ‘timepoint_imaging_v1.ijm’ (for discrete timepoint imaging, for example, Fig. 1d) to generate the necessary tiff image file structures for downstream segmentation and tracking in MATLAB. Our MATLAB timelapse image analysis pipeline then involved the following steps: image alignment (imagealign_barber.m), background removal (to avoid spurious detections, following eraseimagepart_barber.m), followed by cell segmentation and tracking (BacTrack_barber.m). All cell segmentation was done based on phase-contrast images. Once phase-contrast images were analysed to generate individual cell masks (and cell tracks in the case of timelapse imaging), we applied Python scripts to analyse and plot the single cell characteristics, and to integrate fluorescence data (for our discrete timepoint data series). The applicable Python scripts differ based on the purpose and plotting format for individual experiments, and a complete list of the various pipelines for different data formats is provided in the README file in our code repository. Within these steps, we filtered our timelapse cell tracks to exclude cells tracks of 4 timepoints or shorter to exclude non-growing (dead) cells from analysis and to remove sections of cell tracks with a growth rate greater than two standard deviations away from the median growth rate (for example, due to cell divisions or mis-segmentations that combined multiple objects). For our cell staining experiments, we calculated cellular fluorescence by subtracting a background signal (obtained by normalizing a Gaussian blur of the background signal across the cell mask), then calculating the statistics listed on the pixel fluorescence values within individual cell masks. We performed spot detection (for example, for Fig. 1d) with SciKitImage’s bloblog feature using manually curated thresholds for each imaging condition, which were then applied consistently across all images acquired using that condition (including across different cell types).

### MreB filament tracking

We detected and tracked our MreB and Mbl filaments using custom Python scripts as follows: first, we generated our image file structures for downstream analysis using the ImageJ function ‘script tirf_processing.ijm’. We then applied the Python script ‘mreb_tracking_v3_py3.py’, which detected, tracked and classified filaments as follows: for filament detection, we applied SciKitImage’s bloblog feature using manually curated thresholds to start each timelapse (which were then preserved within all timelapses acquired using the same fluorescent fusion protein and imaging condition). These thresholds were iteratively reduced throughout each step of a single timecourse to maintain roughly equivalent levels of spot detection. This allowed us to correct for partial photobleaching of the fluorescent signal. We tracked individual puncta across successive images (taken with a timestep of 2 s) by calculating the minimum distance between all annotated puncta, subject to a maximum displacement threshold of two pixels (equivalent to a linear distance of 186 nm based on our magnification and camera size, significantly greater than the average displacement of a processive individual MreB or Mbl filament of ~80 nm). We then filtered to exclude all tracks with length of 2 timepoints or less. To classify individual tracks as ‘ballistic’, tracks had to meet two criteria. First, we performed a linear regression on a log–log plot of distance (calculated between successive points in each single puncta track and the track’s starting position) versus time. If the slope of this linear regression (the equivalent of ½ the ‘diffusion exponent’ within single-molecule tracking, equal here to 0.5 for diffusive behaviour and 1.0 for ballistic linear motion) was greater than 0.8, ‘and’ if the *R*-value of a linear regression for a standard plot of distances versus time (a metric for the goodness of fit for linear motion) was greater than 0.9, we classified a track as ballistic.

### Cell lysis tracking

We tracked cell lysis by perfusing cells with propidium iodide at a final concentration of 2 μg ml^−1^, imaging every 5 min with 100 ms in the RFP channel at 10% power. Due to a high level of background signal following cell lysis, we annotated cell lysis events using the semi-automated custom ImageJ macros ‘lysis_annotation_lysed_cells_updated.ijm’ and ‘lysis_annotation_plain_cells_updated.ijm’. These macros allowed the user to provide annotations for lysed cells and all cells respectively within each image of a propidium iodide-stained timecourse, which were then integrated and plotted using the Python script ‘lysis_timelapses.py’.

### Analysis of peptidoglycan composition

Peptidoglycan was prepared from cell lysates and analysed by HPLC as previously described^70^: cell lysates were prepared from 500 ml of cell culture by boiling the cells (100 °C) for 30 min in the presence of 5% SDS to inactivate autolysins. The SDS was removed from the insoluble cell wall preparation by repeated centrifugation (130,000*g*, 60 min), the crude cell wall washed with 1 M NaCl and twice with dH_2_O, and resuspended in dH_2_O. The pellet was resuspended in 2–4 ml of dH_2_O, 1/3 volume of acid-washed glass beads (diameter of 0.17–0.18 mm, Sigma) was added, and cell walls were disrupted in a FastPrep machine (FP120, Thermo Scientific). Broken cell walls were separated from the glass beads through filtration and collected by ultracentrifugation as above. Cell walls were incubated at 4 °C with DNase A and RNase (for 2 h) and then with trypsin (18 h), and then incubated with 1% SDS at 80 °C for 15 min. The cell walls were then washed with 8 M LiCl, 20 mM EDTA and water, and lyophilised. The attached wall teichoic acid was removed by treatment with 48% hydrofluoric acid (48 h at 4 °C). The resulting peptidoglycan was recovered by centrifugation, washed once with 50 mM Tris/HCl pH 7.0 and then with ice-cold distilled water until a neutral pH was achieved. The peptidoglycan was digested with cellosyl (gift from Hoechst, Germany), and the resulting muropeptides recovered were reduced with sodium borohydride and separated by HPLC on a 250 × 4.6 mm 3-μm Prontosil 120-3-C_18_ AQ reversed-phase column (Bischoff Analysentechnik) maintained at 52 °C. A 270 min linear gradient reverse-phase separation was performed using 40 mM NaPO_4_ pH 4.5 and 0.003% NaN_3_ (buffer 1, called buffer A in previous work but renamed here to avoid confusion with our GlpQ protein buffer), and 40 mM NaPO_4_ pH 4.0 and 20% methanol (buffer B)^49^. Muropeptides were detected by their absorbance at 202 nm and structures were assigned by comparison to published chromatograms^49^.

### Permeability assays

Cells were grown to log phase and diluted 20-fold into a prewarmed medium within the loading well of a preheated CellASIC B04A microfluidic flow cell. The cells were incubated for an additional 30 min before loading into the imaging chamber. While imaging, fresh medium was perfused through the flow cell. The cell-trapping mechanism used by the microfluidic chips had no detrimental effect on the elongation or morphology of cell chains in comparison to growing on agarose pads or liquid culture^6^.

To perform membrane lysis, we exchanged the medium in the flow cell with media + 5% *N*-lauroylsarcosine (Sigma Aldrich, L9150) for 2 min. We monitored detergent exchange using the dye AlexaFluor 647 at a final media concentration of 5 μg ml^−1^, and imaged cells with CY5 illumination, 640 nm excitation at 10% intensity and 50 ms exposure. After membrane lysis, the detergent in the flow cell was exchanged with media to wash the cells.

Because both tunicamycin and GlpQ are temperature sensitive, all handling steps were performed to better preserve reagent stability: tunicamycin and GlpQ were introduced into the microfluidic device concurrently with the cells. At the end of the culture back-dilution, a 5 mg ml^−1^ tunicamycin stock aliquot was thawed briefly and subjected to two successive 1:100 dilutions in LB before being loaded into the chip. GlpQ aliquots were thawed on ice for ~30 min and diluted in PBS immediately before use. Since PBS-mediated hydrolysis is sensitive to divalent cation concentration, an equal volume of buffer A was diluted in PBS as a control.

For the experiments in Fig. 3a, cells expressing mNeonGreen were imaged every minute in our GFP channel (480 nm excitation from an LED light source, Lumencor Aura Light Engine) at 100% power and 50 ms exposure. To mitigate photobleaching, no images were acquired from 1 min after the onset of tunicamycin treatment to 5 min before detergent exchange. Wild-type and Δ*ponA* cells were exposed to 0.5 µg ml^−1^ tunicamycin for 10 min and 20 min respectively, corresponding to timepoints during which Rod complex activity halts in each strain.

For the experiments in Fig. 3b, cells expressing mNeonGreen were imaged in our GFP channel at 100% power and 50 ms exposure: every minute for 7 min, not acquired for 8 min during buffer A/GlpQ perfusion, every minute for 10 min, and then every 5 min for 1 h. Cells were exposed to GlpQ or buffer A for 15 min before membrane lysis.

For the experiment in Fig. 3c, for cells expressing a tetracysteine-tagged ubiquitin, we imaged in our GFP channel at 10% power and 50 ms exposure every minute for the first 10 min, and every 5 min afterwards. To label the tetracysteine-tagged ubiquitin, we incubated cells with 1 μM FlAsH-EDT_2_ (4’,5’-bis(1,3,2-dithiarsolan-2-yl)-3’,6’-dihydroxy-spiro[isobenzofuran-1(3H),9’-[9H]xanthen]-3-one; Cayman Chemical, 20704) for 1 h before diluting the cells into the loading well, which also had 1 μM FlAsH-EDT_2_ in the medium. To perform membrane lysis and wall teichoic acid hydrolysis simultaneously, the medium in the flow cell was exchanged with PBS + 5% *N*-lauroylsarcosine + GlpQ (or buffer A) for 2 min. We then perfused the cells with PBS + GlpQ (or buffer A). As a control for non-specific binding of FlAsH, we performed the same experiment in buffer A with uninduced cells and subtracted the mean uninduced fluorescence from the fluorescence trace of each cell.

The fluorescence from the frames during lysis were interpolated. The cellular trace was corrected for photobleaching and then normalized to the final pre-lysis frame.

To perform the photobleaching controls^55^, cells were imaged at one frame per minute during the initial 7 min of the experiment before cell lysis. After membrane lysis, the cells were imaged at frame rates that were much more rapid than the rate of protein efflux.

#### Limitations

Our permeability assay can quantitatively assess pores in the cell wall empirically, but cannot resolve the distribution of pore sizes^55^. For example, we could not distinguish between 10 pores that allow the passage of 1 mNeonGreen enzyme at a time, or 1 pore that allows 10 mNeonGreen enzymes to pass through simultaneously. In addition, while we controlled for the effect of the isoelectric point (pI) of the probe, we have not yet explored whether there are other electrical properties of the probe that influence permeability through the wall (for example, the net charge or distribution of acidic/basic residues throughout the protein).

### Permeability photobleaching correction

Population-averaged fluorescence traces were fit to a single exponential:

1 $$y=A{e}^{-x\rho }$$

where *y* is the normalized fluorescence, *A* is the initial post-lysis normalized fluorescence value, *ρ* is the photobleaching constant, and *x* is the frame number.

For mNeonGreen, the mean background value was subtracted from the mean fluorescence of each cell and normalized by the fluorescence of the final pre-lysis frame.

For FlAsH, the mean background value and mean autofluorescence was subtracted from the mean fluorescence of each cell. The mean autofluorescence was obtained by measuring the fluorescence of uninduced cells incubated with FlAsH-EDT_2_.

To correct for photobleaching, the expected loss of fluorescence due to photobleaching was calculated as

2 $${\rm{d}}{C}_{{\rm{B}}}={C}_{i} \rho$$

where *C*_B_ is the bleached fluorophore, *C*_*i*_ is the measured fluorescence in the *i*th frame, and *ρ* is the photobleaching constant (obtained from the photobleaching control). The total loss of fluorescence (d*C*_T_) in each frame was adjusted to account for the expected loss of fluorescence due to photobleaching,

3 $${\rm{d}}{C}_{{\rm{T}}}={C}_{i+1}-{C}_{i}$$

4 $${\rm{d}}{C}_{{\rm{U}}}={\rm{d}}{C}_{{\rm{T}}}+{\rm{d}}{C}_{\rm{B}}$$

5 $${C}_{i+1}={C}_{i}+{\rm{d}}{C}_{{\rm{U}}}$$

where d*C*_U_ is the change in unbleached fluorophore.

### TEM

We performed TEM on thin sections of formaldehyde-fixed freeze-substituted *B. subtilis* cells as follows: we grew cultures of wild-type and Δ*ponA* cells to log phase, then back diluted cultures into either fresh LB medium or LB supplemented with 0.5 μg ml^−1^ tunicamycin for 30 min. We then pelleted cells by centrifugation for 4 min at 3,720*g*, followed by washing 1× in PBS and resuspension in 1 ml freshly made LRR fixative solution^71^ (0.5 ml 0.15% ruthenium red, 125 μl 16% formaldehyde, 0.0155 g lysine acetate (Sigma); distilled water to 1 ml).

Samples were then subjected to high-pressure freezing and freeze substitution. Planchettes (3 mm, 100 mm deep) were lightly coated with hexadecene before being filled with ~1.2 ml of fixed sample. An absorbent filter paper was used to soak out extra liquid, and then the sample was allowed to dry slightly for ~5 min. The hats were then sealed in the planchette holder for high-pressure freezing (Leica ICE High Pressure Freezing Platform, Leica Microsystems). The frozen samples were immediately transferred into liquid nitrogen and then into cryovials containing freeze substitution solutions (2 ml cryovials (Nalgene) containing 2% (w/v) osmium tetroxide (OsO_4_) and 0.1% (w/v) uranyl acetate in anhydrous acetone with 0.075% (w/v) ruthenium red and 2% H_2_O) at liquid nitrogen temperature. The samples were brought into a Leica AFS2 EM freeze substitution unit (Leica Microsystems) and left at −90 °C for 79 h. Since the acetone:osmium mixture liquifies at −90 °C, the hats were slowly submerged in the freeze substitution media. The temperature of the unit was raised 5 °C h^−1^ to −60 °C and incubated for 12 h, then to −30 °C for an additional 6 h, and finally to a temperature of 0 °C for 6 h.

After removal from the freeze substitution unit, samples were washed in pure ethanol on ice 3× for 1 h each time to rinse out osmium. They were then washed with 1:1 100% ethanol:LR white resin on ice for 2 h, 1:2 100% ethanol:LR white resin at 4 °C overnight, pure LR white resin on ice for 8 h, and finally pure LR white resin at 4 °C overnight. Samples were then embedded in gelatin capsules filled with LR white resin at room temperature and polymerized for 48 h at 55–60 °C. Thin sections were cut onto 200 mesh grids and counterstained with 4% aqueous uranyl acetate for 5 min. Stained grids were examined using a JEOL1400 Flash transmission electron microscope and photographed with a Gatan Rio 16 camera. All chemicals and EM grids were purchased from Electron Microscopy Sciences.

Of note, TEM sample preparations of wild type and Δ*ponA* mutants were performed as separate batches, with Δ*ponA* mutant samples being exposed to LRR fixative solution for up to 10 min longer than wild-type cells immediately following lysis. We attribute this difference to the darker cell wall appearance of Δ*ponA* mutants relative to wild-type cells, making it difficult to compare absolute cell wall thickness between these cell types.

### Bootstrap analysis

We performed bootstrap analysis in Fig. 2h by randomly sampling with replacement to generate 100 bootstrapped cell wall thickness samples per condition, then calculating the percentage reduction in thickness for tunicamycin treatment relative to LB. We repeated this 10^5^ times and plotted the average ± s.d. across bootstrapped replicates (standard deviation across bootstrapped replicates approximates the standard error). We tested statistical significance by measuring the percentage of *t*-values below zero across all bootstrapped replicates (equivalent to a one-sided *t*-test). Since this percentage was zero, this indicates statistical significance.

### Statistics and reproducibility

Statistical tests, *P* values and replicate counts are generally listed within figure captions. We have instead included those details here only when necessary due to size constraints. Figure 2a: 621 cell tracks, 5 biological replicates. b: Δ*ponA*: 1,098 cells, 3 biological replicates; wild type: 1,105 cells, 2 biological replicates. c: 4 technical replicates per biological replicate. (1 representative biological replicate of 3 is plotted). d: 6,024 fluorescent tracks, 3 biological replicates. e: 2 biological replicates. f: 32,620 filament tracks, 3 biological replicates.

Figure 3a: wild-type untreated: 87 cells, 4 biological replicates; wild-type tunicamycin: 139 cells, 3 biological replicates; Δ*ponA* untreated: 53 cells, 3 biological replicates; Δ*ponA* tunicamycin: 35 cells, 3 biological replicates. b: wild-type buffer: 121 cells, 1 biological replicate; wild-type GlpQ: 90 cells, 1 biological replicate; Δ*ponA* buffer: 85 cells, 3 biological replicates; Δ*ponA* GlpQ: 108 cells, 3 biological replicates. c: Δ*ponA* buffer: 79 cells, 3 biological replicates; Δ*ponA* GlpQ: 91 cells, 3 biological replicates. d: 1,816 tracks, 3 biological replicates. e: 3,236 cell tracks, 4 biological replicates. f: 3,036 cell tracks, 3 biological replicates. g: wild-type LB: 31 cells; wild-type tunicamycin: 28 cells; Δ*ponA* LB: 47 cells; Δ*ponA* tunicamycin: 44 cells. 1 biological replicate. Significance calculated using two-sided Student’s *t*-test: *p* = 2 × 10^−5^ (WT), *p* = 4 × 10^−10^ (Δ*ponA*). h: significance tested using one-sided Student’s *t*-test, *p* < 0.01 (see Methods: Bootstrap analysis).

Extended Data Fig. 6a: 4 technical replicates per biological replicate, 1 representative biological replicate of 3 is plotted. Significance tested using two-sided Student’s *t*-test, *p* = 3 × 10^−5^. b: 1 representative technical replicate of 2 is shown, 1 biological replicate. c: 3 biological replicates. d: 1,549 fluorescent traces plotted from 1 representative biological replicate of 3. e: 3,320 PBP1 puncta and 6,253 HADA puncta, 13 fields of view, 2 biological replicates. Statistical significance calculated by two-sided Student’s *t*-test, *p* = 5 × 10^−11^. f: 1 representative sample from 3 biological replicates is shown. i: 1 biological replicate.

### Reporting summary

Further information on research design is available in the Nature Portfolio Reporting Summary linked to this article.

## Supplementary information

Supplementary Information Supplementary Figs. 1–5.

Reporting Summary

Supplementary Table 1 Strains, plasmids and primers used in this study.

Supplementary Video 1 ×100 Phase timelapse of wild-type growth during 0.5 μg ml^−1^ tunicamycin treatment. Tunicamycin exposure starts at 5 min; time step, 20 s; scale bar, 5 μm.

Supplementary Video 2 ×100 TIRF imaging of Mbl-sfGFP motion in wild-type cells grown in LB. Time step, 2 s; scale bar, 5 μm.

Supplementary Video 3 ×100 TIRF imaging of Mbl-sfGFP motion in wild-type cells grown in LB. Cells were identically treated to those visualized in Supplementary Video 2. Time step, 2 s; scale bar, 5 μm.

Supplementary Video 4 ×100 TIRF imaging of Mbl-sfGFP motion in wild-type cells grown in LB, then treated with 0.5 μg ml^−1^ tunicamycin for 30 min. Time step, 2 s; scale bar, 5 μm.

Supplementary Video 5 ×100 TIRF imaging of Mbl-sfGFP motion in wild-type cells grown in LB, then treated with 0.5 μg ml^−1^ tunicamycin for 30 min. Cells were identically treated to those visualized in Supplementary Video 4. Time step, 2 s; scale bar, 5 μm.

Supplementary Video 6 ×100 TIRF imaging of Mbl-sfGFP motion in wild-type cells grown in LB, then treated with 0.5 μg ml^−1^ tunicamycin for 60 min. Time step, 2 s; scale bar, 5 μm.

Supplementary Video 7 ×100 TIRF imaging of Mbl-sfGFP motion in wild-type cells grown in LB, then treated with 0.5 μg ml^−1^ tunicamycin for 60 min. Cells were identically treated to those visualized in Supplementary Video 6. Time step, 2 s; scale bar, 5 μm.

Supplementary Video 8 ×100 TIRF imaging of PBP1-mNeonGreen dynamics during steady state growth, driven from a heterologous HyperSpank promoter with 10 μM IPTG in addition to the endogenous copy of *ponA*. Time step, 500 ms; scale bar, 5 μm.

Supplementary Video 9 ×100 TIRF imaging of PBP1-mNeonGreen dynamics, driven from a heterologous HyperSpank promoter with 10 μM IPTG in addition to the endogenous copy of *ponA*. Cells treated with 0.5 μg ml^−1^ tunicamycin for 90 min. Time step, 500 ms; scale bar, 5 μm.

Supplementary Video 10 ×100 TIRF imaging of Mbl-sfGFP motion in Δ*ponA* cells grown in LB supplemented with 10 mM MgCl_2_. Time step, 2 s; scale bar, 5 μm.

Supplementary Video 11 ×100 TIRF imaging of Mbl-sfGFP motion in Δ*ponA* cells grown in LB supplemented with 10 mM MgCl_2_, then treated with 0.5 μg ml^−1^ tunicamycin for 30 min. Time step, 2 s; scale bar, 5 μm.

Supplementary Video 12 ×100 TIRF imaging of Mbl-sfGFP motion in growing wild-type cells treated with 25 μM GlpQ for 15 min. Timelapse taken 25 min post release into LB. Time step, 2 s; scale bar, 5 μm.

Supplementary Video 13 ×100 TIRF imaging of Mbl-sfGFP motion in growing wild-type cells treated with 25 μM GlpQ for 15 min. Timelapse taken 35 min post release into LB. Time step, 2 s; scale bar, 5 μm.

Supplementary Video 14 ×100 TIRF imaging of Mbl-sfGFP in non-growing wild-type cells treated with 25 μM GlpQ for 15 min. Timelapse taken 25 min post release into LB. Time step, 2 s; scale bar, 5 μm.

Supplementary Video 15 ×100 TIRF imaging of Mbl-sfGFP in non-growing wild-type cells treated with 25 μM GlpQ for 15 min. Timelapse taken 35 min post release into LB. Time step, 2 s; scale bar, 5 μm.

## Source data

Source Data Fig. 1 Unmodified western blots and gels.

## Data Availability

All datasets presented herein are publicly available in figshare at 10.6084/m9.figshare.c.8406249 (ref. ^72^). Raw data for uncropped immunoblots are provided as Source data with this paper.

## Code availability

All code used here is publicly available in Zenodo at 10.5281/zenodo.19461850 (ref. ^73^).
