# Supplementary material for: Wall teichoic acids regulate peptidoglycan synthesis to maintain rod shape in Bacillus subtilis
Source: Nat Microbiol. 2026 May 26;11(7):1893–906. doi: 10.1038/s41564-026-02368-6 (PMC13270295; doi:10.1038/s41564-026-02368-6)
Supplement: Supplementary file 1 — Supplementary Figs. 1–5. [file 41564_2026_2368_MOESM1_ESM.pdf]

nature microbiology

Supplementary information

https://doi.org/10.1038/s41564-026-02368-6

Wall teichoic acids regulate peptidoglycan synthesis to maintain rod shape in *Bacillus subtilis*

In the format provided by the authors and unedited

Supplemental Figures:

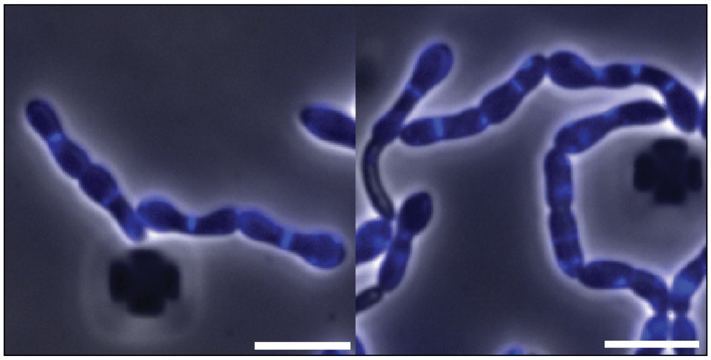

Figure S1: Cell bulging occurs gradually a "dumbbell" phenotype during teichoic acid depletion. Cell wall bulges qualitatively became more prevalent with increasing distance from internal septa. Micrographs show HADA fluorescent staining in blue, superimposed over phase contrast images of cells following 45 min exposure to 0.5μg/mL tunicamycin and 5min HADA incubation. Scale bars 5μm. One biological replicate.

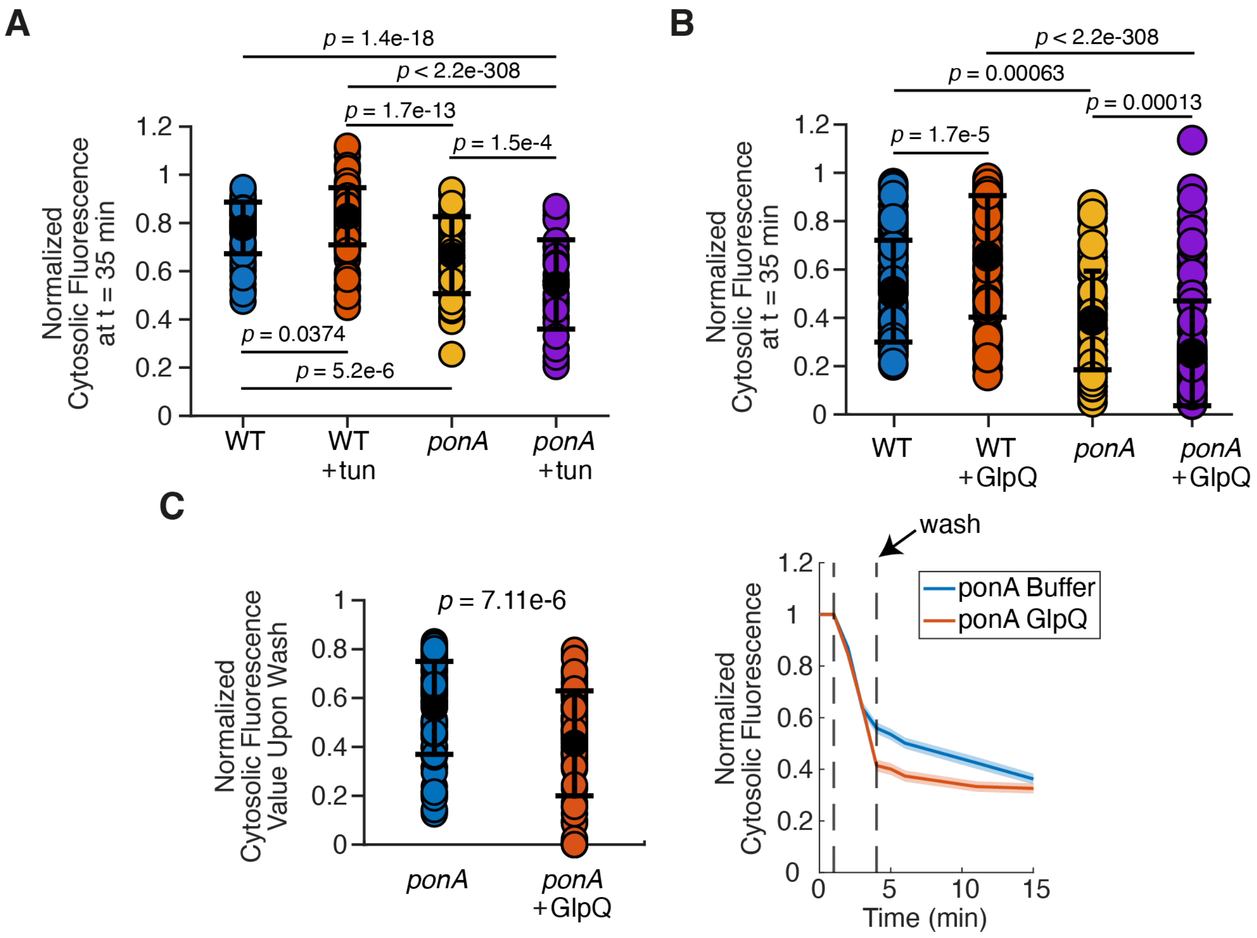

Figure S2: Statistical significance and decay statistics for cell wall permeability assay. (A) mNeonGreen fluorescence at t=35 min for wild-type and Δ*ponA* cells either treated with tunicamycin or untreated. Data are plotted as average across cells +/- standard deviation (individual cells as dots). Statistical significance determined via one-way ANOVA (*p* = 1.9e-27) followed by Tukey's HSD post-hoc test for multiple comparisons with *p* = 0.04 (WT, WT + tun), *p* = 2e-4 (Δ*ponA*, Δ*ponA* + tunicamycin), *p* = 5e-6 (WT, Δ*ponA*), *p* = 2e-13 (WT + tun, Δ*ponA*), *p* = 1e-18 (WT, Δ*ponA* + tun), *p* < 2e-308 (WT + tun, Δ*ponA* + tun). Wild-type untreated: 87 cells, 4 biological replicates. Wild-type tunicamycin: 139 cells, 3 biological replicates. Δ*ponA* untreated: 53 cells, 3 biological replicates. Δ*ponA* tunicamycin: 35 cells, 3 biological replicates. (B) mNeonGreen fluorescence at t=35 min for wild-type and Δ*ponA* cells either treated with GlpQ in PBS or PBS alone. Data are plotted as average across cells +/- standard deviation (individual cells as dots).Statistical significance determined via one-way ANOVA (*p* = 8.7e-32) followed by Tukey's HSD post-hoc test for multiple comparisons with *p* = 2e-5 (WT, WT + GlpQ), *p* = 1e-4 (Δ*ponA*, Δ*ponA* + GlpQ), *p* = 6e-4 (WT, Δ*ponA*), *p* < 2e-308 (WT + GlpQ, Δ*ponA* + GlpQ), *p* = 3e-18 (WT, Δ*ponA* + GlpQ), *p* = 1e-14 (WT + GlpQ, Δ*ponA*). Wild-type Buffer:

121 cells, 1 biological replicate. Wild-type GlpQ: 90 cells, 1 biological replicate. Δ*ponA* Buffer: 85 cells, 3 biological replicates. Δ*ponA* GlpQ: 108 cells, 3 biological replicates. (C) mUbiquitin fluorescence upon wash (as indicated by the arrow on the fluorescence vs time graph, right) following incubation of Δ*ponA* cells with detergent +/- GlpQ. Data are plotted as average across cells +/- standard deviation (individual cells as dots). Statistical significance calculated using two-sided Student's *t*-test, p = 7e-6. Δ*ponA* Buffer: 79 cells, three biological replicates. Δ*ponA* GlpQ: 91 cells, three biological replicates.

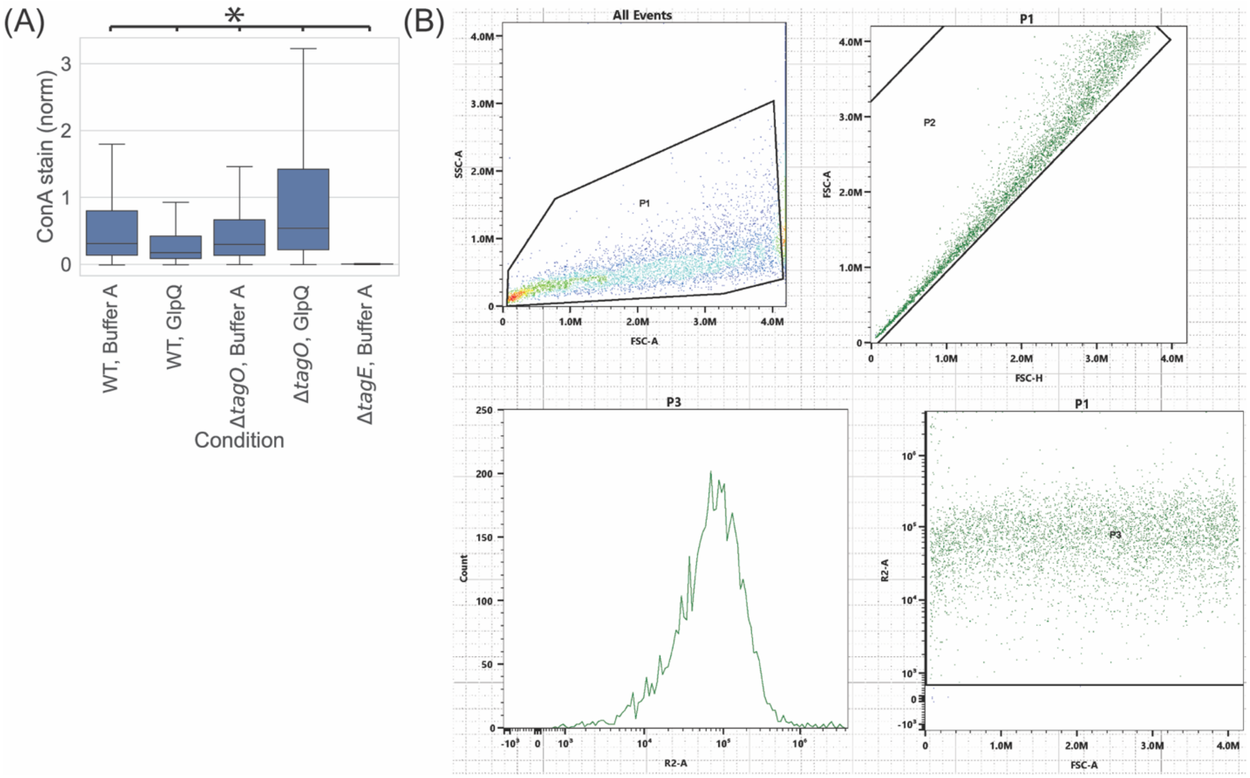

Figure S3: Concanavalin A staining decreases following GlpQ treatment. (A) Box plots showing fluorescence intensity of Concanavalin A-AlexaFluor647 cell staining, following incubation either with PBS + Buffer A, or with PBS + GlpQ, measured by flow cytometry. Box lines show quartiles, whiskers show spread of data (excluding outliers for ease of presentation). GlpQ-treated cells show decreased Concanavalin A staining relative to PBS-treated cells, consistent with GlpQ enzymatically cleaving teichoic acids from the cell wall. Δ*tagO* cells show no such decrease in Concanavalin A staining upon GlpQ incubation, and Δ*tagE* cells lacking the enzyme responsible for teichoic acid glycosylation show negligible labeling. We observed increased Concanavalin A-staining in Δ*tagO* cells relative to wild-type that was amplified during GlpQ treatment, possibly due to the clumping phenotype of this mutant. n=50,000 (WT, Buffer A — 5 biological replicates), n=40,000 (WT, GlpQ — 4 biological replicates), n=30,000 (Δ*tagO*, Buffer A — 3 biological replicates), n=30,000 (Δ*tagO*, GlpQ — 3 biological replicates), n=30,000 (Δ*tagE*, Buffer A _— 3 biological replicates). 10,000 samples per replicate. All conditions show statistically significant differences as measured by one-way ANOVA followed by Tukey's HSD post-hoc test, *p*-value below computational detection limit. **(B)** Representative example of flow cytometry gating process used to generate data in (A).

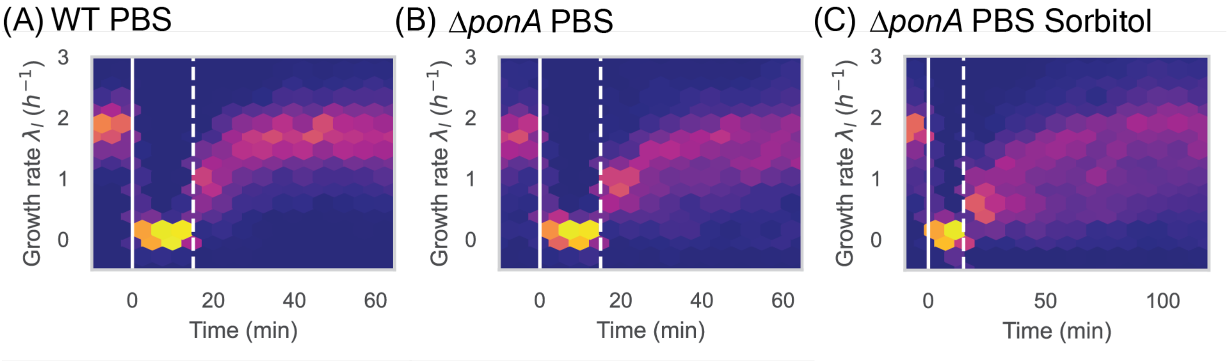

Figure S4: Cell growth uniformly recovers following PBS treatment. (A-B) Heat maps showing growth rate $\lambda_l = \frac{1}{l}\frac{dl}{dt}$ during recovery after incubation with either PBS + denatured GlpQ or GlpQ buffer for 15 min. **(A)** wild-type cells (3,830 discrete tracks, two independent experiments with denatured GlpQ, one with equivalent volume GlpQ buffer, no differences between conditions observed). **(B)** Δ*ponA* cells (2,142 discrete cell tracks, 3 biological replicates). **(C)** Heatmap showing Δ*ponA* cell length growth rate $\lambda_l = \frac{1}{l}\frac{dl}{dt}$ following 15-minute incubation with PBS + GlpQ Buffer (*not* GlpQ enzyme) (7,097 discrete cell tracks, 4 biological replicates), where exit from incubation is coupled to a 500mM Sorbitol hyperosmotic shock. Solid line shows onset of PBS incubation, dotted line shows exit into LB + Sorbitol.

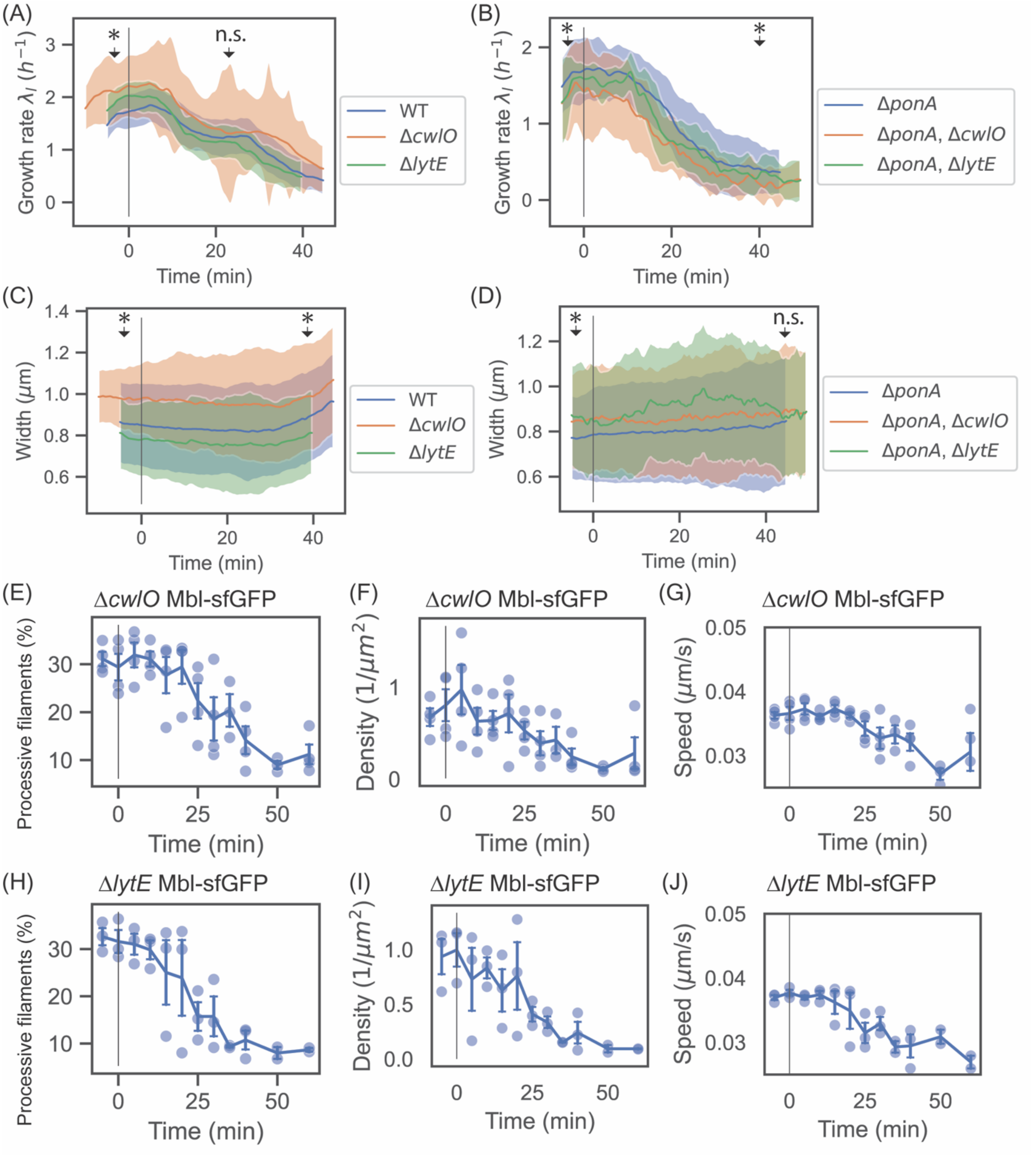

Figure S5. Elongation decline, Rod complex arrest and shape loss occur during wall teichoic acid depletion in Δ*cwlO* and Δ*lytE* mutants. (A-B) Cell elongation rate during tunicamycin treatment for **(A)** wild-type, Δ*cwlO* and Δ*lytE* cells and **(B)** Δ*ponA*, Δ*ponA* Δ*cwlO* and Δ*ponA* Δ*lytE* cells. Solid lines show smoothed population medians, error bars show standard deviations across cells. **(A)** Strains showed statistically significant differences in initial growth rate (time=-5min), but converged to a plateau growth

rate that did not reject the null (equal) hypothesis (t=22 min following tunicamycin treatment). Significance calculated using two-sided Student's *t*-test. Time = -5min: *p* = 1e-40 (WT v. Δ*cwlO*), *p* = 6e-16 (WT v. Δ*lytE*), *p* = 1e-4 (Δ*lytE* v. Δ*cwlO*). Time = 22min: *p* = 0.3 (WT v. Δ*cwlO*), *p* = 0.01 (WT v. Δ*lytE*), *p* = 0.6 (Δ*lytE* v. Δ*cwlO*). Analysis performed over 1,091 discrete cell tracks from 3 experimental replicates (Δ*cwlO*), and 1,277 discrete cell tracks plotted from 3 experimental replicates (Δ*lytE*). Wild-type data replotted from Fig. 1B. **(B)** Hydrolase mutants showed significantly lower initial growth rates than the Δ*ponA* mutant (first measured timepoint), while the Δ*ponA* Δ*cwlO* mutant showed a significantly lower final growth rate than the Δ*ponA* mutant. All strains lacked the plateau in growth rate of wild-type cells. Analysis performed over 164 discrete cell tracks from two experimental replicates (Δ*ponA* Δ*cwlO*), and 105 discrete cell tracks plotted from two experimental replicates (Δ*ponA* Δ*lytE*). Δ*ponA* data replotted from Fig. 2A. Significance tested using two-sided Student's *t*-test at the first and last measured timepoints. Time = -5min: *p* = 3e-8 (Δ*ponA* v. Δ*cwlO* Δ*ponA*), *p* = 1e-6 (Δ*ponA* v. Δ*lytE* Δ*ponA*), *p* = 0.6 (Δ*lytE* Δ*ponA* v. Δ*cwlO* Δ*ponA*). Time = 45min: *p* = 6e-5 (Δ*ponA* v. Δ*cwlO* Δ*ponA*), *p* = 0.02 (Δ*ponA* v. Δ*lytE* Δ*ponA*), *p* = 0.08 (Δ*lytE* Δ*ponA* v. Δ*cwlO* Δ*ponA*). **(C-D)** Cell width dynamics during tunicamycin treatment for **(C)** wild-type, Δ*cwlO* and Δ*lytE* cells and **(D)** Δ*ponA*, Δ*ponA* Δ*cwlO* and Δ*ponA* Δ*lytE* cells. Solid lines show smoothed population medians, error bars show standard deviations across cells. **(C)** Strains showed statistically significant differences in width throughout, calculated using two-sided Student's *t*-test at the first and last measured timepoints. Time = -5min: *p* = 7e-10 (WT v. Δ*cwlO*), *p* = 5e-4 (WT v. Δ*lytE*), *p* = 2e-18 (Δ*lytE* v. Δ*cwlO*). Time = 40min: *p* = 1e-15 (WT v. Δ*cwlO*), *p* = 4e-13 (WT v. Δ*lytE*), *p* = 8e-36 (Δ*lytE* v. Δ*cwlO*). All strains display characteristic cell widening at long times. Analysis performed over 1,746 discrete cell tracks from 3 experimental replicates (Δ*cwlO*), and 2,432 discrete cell tracks from 3 experimental replicates (Δ*lytE*). Wild-type data replotted from Fig. 1B. **(D)** Hydrolase mutants showed statistically significant differences in width from Δ*ponA* mutant at the first measured timepoint, but showed no significant differences at the final timepoint (t=45 min tunicamycin exposure) and consistently lacked the characteristic cell widening of wild-type cells at long times. Significance tested using two-sided Student's *t*-test at the first and last measured timepoints. Time = -5min: *p* = 3e-4 (Δ*ponA* v. Δ*cwlO* Δ*ponA*), *p* = 9e-9 (Δ*ponA* v. Δ*lytE* Δ*ponA*), *p* = 0.07 (Δ*lytE* Δ*ponA* v. Δ*cwlO* Δ*ponA*). Time = 45min: *p* = 0.5 (Δ*ponA* v. Δ*cwlO* Δ*ponA*), *p* = 0.3 (Δ*ponA* v. Δ*lytE* Δ*ponA*), *p* = 0.2 (Δ*lytE* Δ*ponA* v. Δ*cwlO* Δ*ponA*). Analysis performed over 1,747 discrete cell tracks from five biological replicates (Δ*ponA*), 632 discrete cell tracks from two experimental replicates (Δ*ponA* Δ*cwlO*), and 585 discrete cell tracks from two experimental replicates (Δ*ponA* Δ*lytE*). **(E-J)** Rod complex dynamics measured by tracking Mbl-sfGFP filaments during tunicamycin treatment for **(E-G)** Δ*cwlO* and **(H-J)** Δ*lytE* cells. Data are presented as average across biological

replicates +/- SEM, with individual biological replicates as dots. **(E, H)** Percentage of processive filaments. **(F, I)** Spatial density of processive filaments. **(G, J)** Average speed of processive filaments. Analysis performed over 37,707 tracked filaments from four biological replicates (Δ*cwlO*) and 45,748 tracked filaments from three biological replicates (Δ*lytE*).
