# Supplementary material for: Wall teichoic acids regulate peptidoglycan synthesis to maintain rod shape in Bacillus subtilis
Source: Nat Microbiol. 2026 May 26;11(7):1893–906. doi: 10.1038/s41564-026-02368-6 (PMC13270295; doi:10.1038/s41564-026-02368-6)
Supplement: Supplementary file 2 — Reporting Summary [file 41564_2026_2368_MOESM2_ESM.pdf]

nature portfolio

Corresponding author(s): Enrique Rojas

Last updated by author(s): 4/8/2026

Reporting Summary

Nature Portfolio wishes to improve the reproducibility of the work that we publish. This form provides structure for consistency and transparency in reporting. For further information on Nature Portfolio policies, see our Editorial Policies and the Editorial Policy Checklist.

Statistics

For all statistical analyses, confirm that the following items are present in the figure legend, table legend, main text, or Methods section.

n/a	Confirmed	
☐	☒	The exact sample size (*n*) for each experimental group/condition, given as a discrete number and unit of measurement
☐	☒	A statement on whether measurements were taken from distinct samples or whether the same sample was measured repeatedly
☐	☒	The statistical test(s) used AND whether they are one- or two-sided<br>*Only common tests should be described solely by name; describe more complex techniques in the Methods section.*
☒	☐	A description of all covariates tested
☐	☒	A description of any assumptions or corrections, such as tests of normality and adjustment for multiple comparisons
☐	☒	A full description of the statistical parameters including central tendency (e.g. means) or other basic estimates (e.g. regression coefficient) AND variation (e.g. standard deviation) or associated estimates of uncertainty (e.g. confidence intervals)
☐	☒	For null hypothesis testing, the test statistic (e.g. *F*, *t*, *r*) with confidence intervals, effect sizes, degrees of freedom and *P* value noted<br>*Give P values as exact values whenever suitable.*
☒	☐	For Bayesian analysis, information on the choice of priors and Markov chain Monte Carlo settings
☒	☐	For hierarchical and complex designs, identification of the appropriate level for tests and full reporting of outcomes
☒	☐	Estimates of effect sizes (e.g. Cohen's *d*, Pearson's *r*), indicating how they were calculated

Our web collection on statistics for biologists contains articles on many of the points above.

Software and code

Policy information about availability of computer code

Data collection	Data collection was performed using NIS-Elements, Cytek SpectroFlo, Cytiva Amersham Typhoon control software, and Tecan iControl software.
Data analysis	We used custom scripts to analyze our data that were written in python, MATLAB and FIJI. All software is available for download at: DOI: 10.5281/zenodo.19461851 . Version 1.0.

For manuscripts utilizing custom algorithms or software that are central to the research but not yet described in published literature, software must be made available to editors and reviewers. We strongly encourage code deposition in a community repository (e.g. GitHub). See the Nature Portfolio guidelines for submitting code & software for further information.

Data

Policy information about availability of data

All manuscripts must include a data availability statement. This statement should provide the following information, where applicable:

- Accession codes, unique identifiers, or web links for publicly available datasets
- A description of any restrictions on data availability
- For clinical datasets or third party data, please ensure that the statement adheres to our policy

All datasets presented herein are available publicly at https://doi.org/10.6084/m9.figshare.c.8406249. Raw data for uncropped immunoblots have been uploaded as source data.

Research involving human participants, their data, or biological material

Policy information about studies with human participants or human data. See also policy information about sex, gender (identity/presentation), and sexual orientation and race, ethnicity and racism.

Reporting on sex and gender	N/A.
Reporting on race, ethnicity, or other socially relevant groupings	N/A.
Population characteristics	N/A.
Recruitment	N/A.
Ethics oversight	N/A.

Note that full information on the approval of the study protocol must also be provided in the manuscript.

Field-specific reporting

Please select the one below that is the best fit for your research. If you are not sure, read the appropriate sections before making your selection.

☒ Life sciences ☐ Behavioural & social sciences ☐ Ecological, evolutionary & environmental sciences

For a reference copy of the document with all sections, see nature.com/documents/nr-reporting-summary-flat.pdf

Life sciences study design

All studies must disclose on these points even when the disclosure is negative.

Sample size	We presented the maximal number of practically obtainable datapoints in each experiment, based on experimental limitations and given the biological replicate numbers listed.
Data exclusions	To avoid tracking debris, we filtered out non-growing items from our automated image analysis.
Replication	Wherever practically feasible, we performed multiple (ideally three) biological replicates on each experiment. Within the text, we either presented data from all experiments or showed data representative of all replicates. All experimental findings presented herein were successfully reproduced.
Randomization	Randomization was not necessary since our experiments employ identically prepared, clonal populations of cells.
Blinding	Investigators were not blinded, but blinding was not necessary since our analysis pipelines are automated and therefore faithfully reflect underlying differences in the data irrespective of user bias.

Reporting for specific materials, systems and methods

We require information from authors about some types of materials, experimental systems and methods used in many studies. Here, indicate whether each material, system or method listed is relevant to your study. If you are not sure if a list item applies to your research, read the appropriate section before selecting a response.

Materials & experimental systems

n/a	Involved in the study	
☐	☒	Antibodies
☒	☐	Eukaryotic cell lines
☒	☐	Palaeontology and archaeology
☐	☒	Animals and other organisms
☒	☐	Clinical data
☒	☐	Dual use research of concern
☒	☐	Plants

Methods

n/a	Involved in the study	
☒	☐	ChIP-seq
☐	☒	Flow cytometry
☒	☐	MRI-based neuroimaging

Antibodies

Antibodies used	DYKDDDDK Tag Monoclonal Antibody (2500X dilution, GenScript A01868-40 — lot # not recorded), Goat anti-Rabbit IgG (H+L) Highly

Antibodies used	Cross-Adsorbed Secondary Antibody, Alexa Fluor™ 647 (2500X dilution, Invitrogen, A-21245 — lot # not recorded), rabbit IgG anti-sigA (10,000X dilution, gift from the laboratory of Fujita Masaya)
Validation	A01868-40: The rabbit immune system generates antibody diversity and optimizes affinity. GenScript utilizes MonoRab™ technology to generate the high affinity and specificity monoclonal rabbit antibodies. GenScript MonoRab™ DYKDDDDK Tag Antibody, mAb, Rabbit specific to DYKDDDDK tags placed at C-terminal, N-terminal and internal regions of fusion proteins. The antibody can greatly improve the effectiveness of several different kinds of immunoassays, helping researchers identify, detect, and purify DYKDDDDK fusion proteins in bacteria and mammalian cells.<br><br>A-21245: To minimize cross-reactivity, these goat anti-rabbit IgG whole antibodies have been cross-adsorbed against bovine IgG, goat IgG, mouse IgG, rat IgG, and human IgG. Cross-adsorption or pre-adsorption is a purification step to increase specificity of the antibody resulting in higher sensitivity and less background staining. The secondary antibody solution is passed through a column matrix containing immobilized serum proteins from potentially cross-reactive species. Only the nonspecific-binding secondary antibodies are captured in the column, and the highly specific secondaries flow through. The benefits of this extra step are apparent in multiplexing/multicolor-staining experiments (e.g., flow cytometry) where there is potential cross-reactivity with other primary antibodies or in tissue/cell fluorescent staining experiments where there are may be the presence of endogenous immunoglobulins.<br><br>rabbit IgG anti-sigA: Genes Cells 2000 Feb;5(2):79-88.<br>Temporal and selective association of multiple sigma factors with RNA polymerase during sporulation in Bacillus subtilis<br>PMID: 10672039<br>DOI: 10.1046/j.1365-2443.2000.00307.x<br>Immunodetection was carried out using the monospecific antibodies against each protein and horseradish peroxide-labelled anti-rabbit IgG anti-serum (Wako). Antibody against core RNA polymerase was used for the detection of alpha and core RNA polymerase (Fujita & Sadaie 1998c).

Animals and other research organisms

Policy information about studies involving animals; ARRIVE guidelines recommended for reporting animal research, and Sex and Gender in Research

Laboratory animals	We performed our experiments with Bacillus subtilis bacteria from a PY79 genetic background. All strains are listed and will be made available upon request.
Wild animals	N/A
Reporting on sex	N/A
Field-collected samples	N/A
Ethics oversight	N/A

Note that full information on the approval of the study protocol must also be provided in the manuscript.

Plants

Seed stocks	N/A
Novel plant genotypes	N/A
Authentication	N/A

Flow Cytometry

Plots

Confirm that:

☒ The axis labels state the marker and fluorochrome used (e.g. CD4-FITC).

☒ The axis scales are clearly visible. Include numbers along axes only for bottom left plot of group (a 'group' is an analysis of identical markers).

☒ All plots are contour plots with outliers or pseudocolor plots.

☐ A numerical value for number of cells or percentage (with statistics) is provided.

Methodology

Sample preparation	We grew cell cultures to log-phase (OD600=0.1-0.2), then took two samples from a single cell culture, washed 1X in PBS, and incubated in PBS + 40µM GlpQ or PBS + the equivalent volume of Buffer A for 15 min with rocking at 37°C. We then washed cells again 1X in PBS, and resuspended cells in PBS supplemented with 200ug/mL of Concanavalin A-AlexaFluor 647 (Invitrogen, C21421) for 10min at 37°C, with rocking. Concanavalin A is a lectin that binds glycosylation modifications along the teichoic acid polymer5,6. We then washed cells again 1X in PBS, then resuspended samples in PBS for flow cytometry. We performed our flow cytometry measurements with a Cytek Aurora set to 10,000 events per sample, with gains adjusted to capture the full dynamic range of our population measurements. We consistently calibrated the instrument to a low background (<10 events/second), and vigorously vortexed each sample prior to acquisition. Since we were interested in statistics of the whole cell population, we performed minimal gating (Fig. S6B). Since GlpQ preferentially cleaves non-glycosylated teichoic acids2, the GlpQ-mediated reduction in Concanavalin A staining for wild-type cells is likely an underestimate of GlpQ-mediated teichoic acid cleavage. We used ΔtagE mutants to control for non-specific wheat-germ agglutinin labeling, since TagE is responsible for teichoic acid glycosylation7.
Instrument	Cytek Aurora
Software	Cytiva SpectroFlo, combined with custom data analysis in python.
Cell population abundance	All replicates used 10,000 cells pre-gating.
Gating strategy	Minimal selective gating was applied, since we were interested in whole-population-level statistics.

☒ Tick this box to confirm that a figure exemplifying the gating strategy is provided in the Supplementary Information.
