# Supplementary figures and images for: Wall teichoic acids regulate peptidoglycan synthesis to maintain rod shape in Bacillus subtilis
Source: Nat Microbiol. 2026 May 26;11(7):1893–906. doi: 10.1038/s41564-026-02368-6 (PMC13270295; doi:10.1038/s41564-026-02368-6)

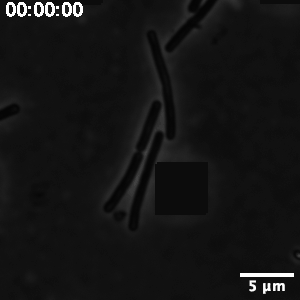

Supplement: Supplementary file 4 — ×100 Phase timelapse of wild-type growth during 0.5 μg ml−1 tunicamycin treatment. Tunicamycin exposure starts at 5 min; time step, 20 s; scale bar, 5 μm. [file 41564_2026_2368_MOESM4_ESM.gif]

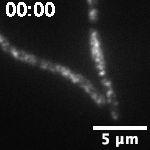

Supplement: Supplementary file 5 — ×100 TIRF imaging of Mbl-sfGFP motion in wild-type cells grown in LB. Time step, 2 s; scale bar, 5 μm. [file 41564_2026_2368_MOESM5_ESM.gif]

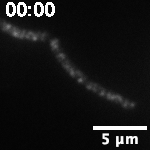

Supplement: Supplementary file 6 — ×100 TIRF imaging of Mbl-sfGFP motion in wild-type cells grown in LB. Cells were identically treated to those visualized in Supplementary Video 2. Time step, 2 s; scale bar, 5 μm. [file 41564_2026_2368_MOESM6_ESM.gif]

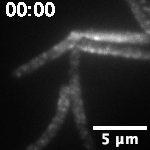

Supplement: Supplementary file 7 — ×100 TIRF imaging of Mbl-sfGFP motion in wild-type cells grown in LB, then treated with 0.5 μg ml−1 tunicamycin for 30 min. Time step, 2 s; scale bar, 5 μm. [file 41564_2026_2368_MOESM7_ESM.gif]

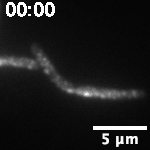

Supplement: Supplementary file 8 — ×100 TIRF imaging of Mbl-sfGFP motion in wild-type cells grown in LB, then treated with 0.5 μg ml−1 tunicamycin for 30 min. Cells were identically treated to those visualized in Supplementary Video 4. Time step, 2 s; scale bar, 5 μm. [file 41564_2026_2368_MOESM8_ESM.gif]

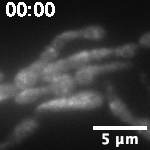

Supplement: Supplementary file 9 — ×100 TIRF imaging of Mbl-sfGFP motion in wild-type cells grown in LB, then treated with 0.5 μg ml−1 tunicamycin for 60 min. Time step, 2 s; scale bar, 5 μm. [file 41564_2026_2368_MOESM9_ESM.gif]

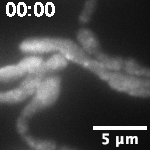

Supplement: Supplementary file 10 — ×100 TIRF imaging of Mbl-sfGFP motion in wild-type cells grown in LB, then treated with 0.5 μg ml−1 tunicamycin for 60 min. Cells were identically treated to those visualized in Supplementary Video 6. Time step, 2 s; scale bar, 5 μm. [file 41564_2026_2368_MOESM10_ESM.gif]

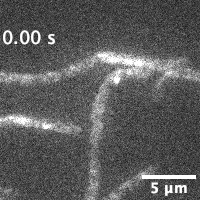

Supplement: Supplementary file 11 — ×100 TIRF imaging of PBP1-mNeonGreen dynamics during steady state growth, driven from a heterologous HyperSpank promoter with 10 μM IPTG in addition to the endogenous copy of ponA. Time step, 500 ms; scale bar, 5 μm. [file 41564_2026_2368_MOESM11_ESM.gif]

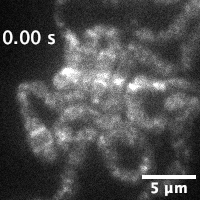

Supplement: Supplementary file 12 — ×100 TIRF imaging of PBP1-mNeonGreen dynamics, driven from a heterologous HyperSpank promoter with 10 μM IPTG in addition to the endogenous copy of ponA. Cells treated with 0.5 μg ml−1 tunicamycin for 90 min. Time step, 500 ms; scale bar, 5 μm. [file 41564_2026_2368_MOESM12_ESM.gif]

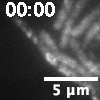

Supplement: Supplementary file 13 — ×100 TIRF imaging of Mbl-sfGFP motion in ΔponA cells grown in LB supplemented with 10 mM MgCl2. Time step, 2 s; scale bar, 5 μm. [file 41564_2026_2368_MOESM13_ESM.gif]

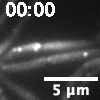

Supplement: Supplementary file 14 — ×100 TIRF imaging of Mbl-sfGFP motion in ΔponA cells grown in LB supplemented with 10 mM MgCl2, then treated with 0.5 μg ml−1 tunicamycin for 30 min. Time step, 2 s; scale bar, 5 μm. [file 41564_2026_2368_MOESM14_ESM.gif]

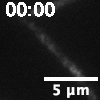

Supplement: Supplementary file 15 — ×100 TIRF imaging of Mbl-sfGFP motion in growing wild-type cells treated with 25 μM GlpQ for 15 min. Timelapse taken 25 min post release into LB. Time step, 2 s; scale bar, 5 μm. [file 41564_2026_2368_MOESM15_ESM.gif]

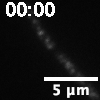

Supplement: Supplementary file 16 — ×100 TIRF imaging of Mbl-sfGFP motion in growing wild-type cells treated with 25 μM GlpQ for 15 min. Timelapse taken 35 min post release into LB. Time step, 2 s; scale bar, 5 μm. [file 41564_2026_2368_MOESM16_ESM.gif]

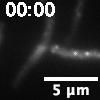

Supplement: Supplementary file 17 — ×100 TIRF imaging of Mbl-sfGFP in non-growing wild-type cells treated with 25 μM GlpQ for 15 min. Timelapse taken 25 min post release into LB. Time step, 2 s; scale bar, 5 μm. [file 41564_2026_2368_MOESM17_ESM.gif]

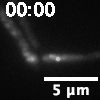

Supplement: Supplementary file 18 — ×100 TIRF imaging of Mbl-sfGFP in non-growing wild-type cells treated with 25 μM GlpQ for 15 min. Timelapse taken 35 min post release into LB. Time step, 2 s; scale bar, 5 μm. [file 41564_2026_2368_MOESM18_ESM.gif]
