# Supplementary material for: Wall teichoic acids regulate peptidoglycan synthesis to maintain rod shape in Bacillus subtilis
Source: Nat Microbiol. 2026 May 26;11(7):1893–906. doi: 10.1038/s41564-026-02368-6 (PMC13270295; doi:10.1038/s41564-026-02368-6)

Source Data Fig. 1

Source Data for Figure 2E. Top membrane: rabbit anti-FLAG, goat anti-rabbit AF647. Bottom membrane: rabbit anti-sigA, goat anti-rabbit AF647. Wells: Ladder, 0min tun. *ponA-FLAG* (bMD643), 10min tun. *ponA-FLAG*, 20min tun. *ponA-FLAG*, 30min tun. *ponA-FLAG*, 40min tun. *ponA-FLAG*, 60min tun. *ponA-FLAG*, *pbpA-FLAG* LB (bMD640). Membranes were identically loaded and processed in parallel.

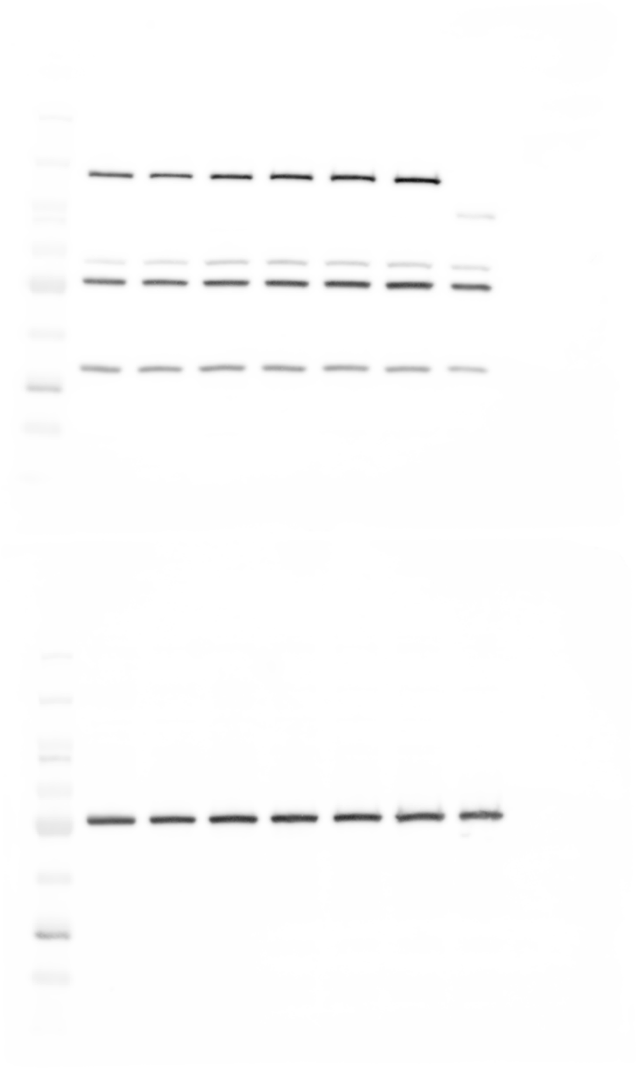

Source Data Fig. 2

Source Data for Extended Data Fig. 6B. SDS-PAGE gel with cell lysates from cultures incubated with fluorescently conjugated bocillin. Top: Cy2 fluorescent imaging. Bottom: Cy5 ladder imaging. Wells: Ladder, wild-type, $\Delta ponA$, $ponA_{\Delta IDR}$, $ponA_{GT-}$, $ponA_{TP-}$.

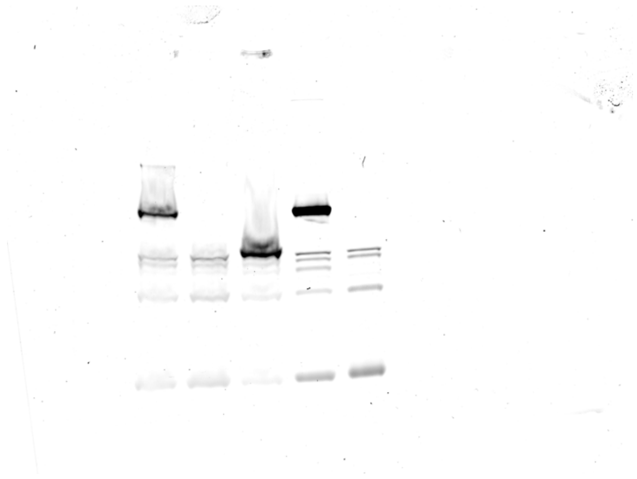

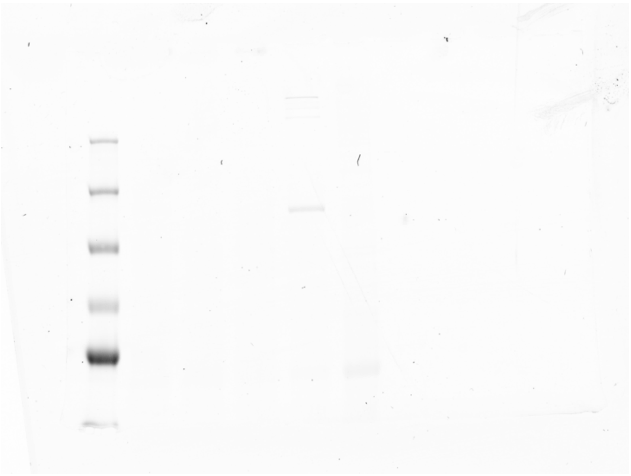

Source Data Fig. 3

Source Data for Extended Data Fig. 6F. Wells: Ladder, 0 min tun. WT, 5 min tun. WT, 10 min tun. WT, 20 min tun. WT, 30 min tun. WT, 40 min tun. WT, WT LB, Δ*ponA* LB, *ponA*$_{TP-}$ LB.

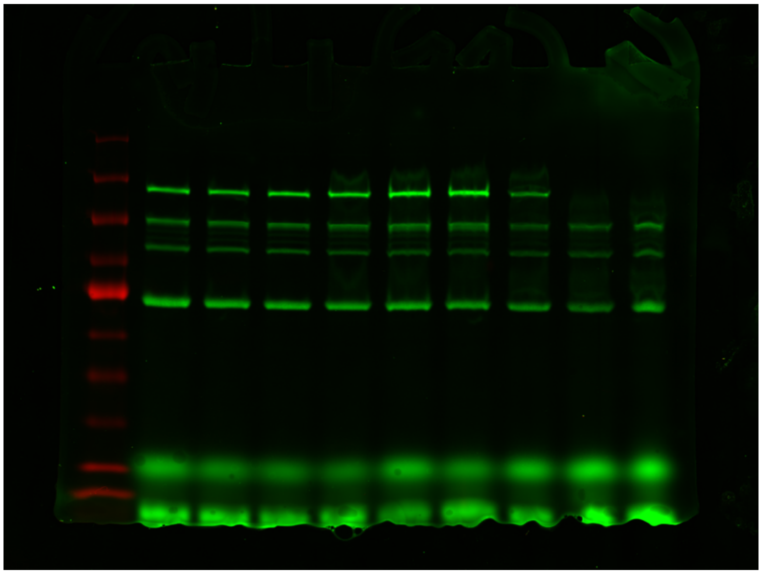

Source Data Fig. 4:

Unprocessed western blots for Extended Data Fig. 6I. Top membrane: rabbit anti-FLAG, goat anti-rabbit AF647. Bottom membrane: rabbit anti-sigA, goat anti-rabbit AF647. Wells: Ladder, 0min tun. *ponA-FLAG* Δ*sigI*, 10min tun. *ponA-FLAG* Δ*sigI*, 20min tun. *ponA-FLAG* Δ*sigI*, 30min tun. *ponA-FLAG* Δ*sigI*, 40min tun. *ponA-FLAG* Δ*sigI*, 60min tun. *ponA-FLAG* Δ*sigI*. Membranes were identically loaded and processed in parallel.

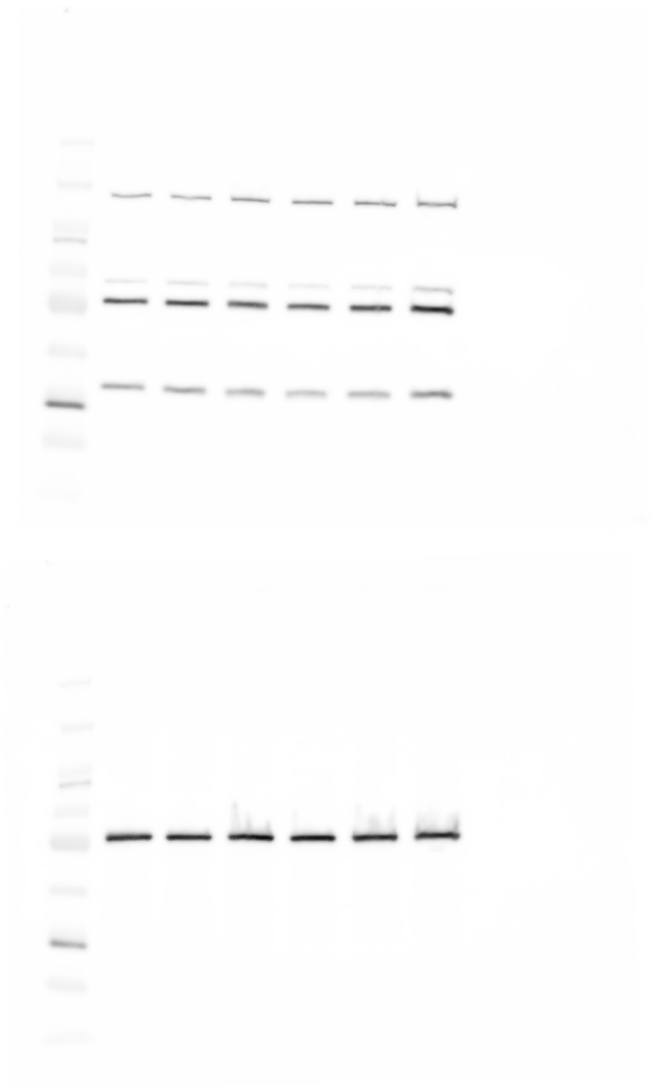

Supplement: Supplementary file 19 — Unmodified western blots and gels. [file 41564_2026_2368_MOESM19_ESM.pdf]
